# Active‐Site Interactions in a Synergistic Porous Structured Fe Nanoparticle–Carbon Electrocatalyst for Enhanced Redox Reactions in Alkaline Zn–Air Batteries

**DOI:** 10.1002/smsc.202500448

**Published:** 2026-02-13

**Authors:** Ramasamy Santhosh Kumar, Pandian Mannu, Venkatesan Srinivasadesikan, Narayanamoorthy Bhuvanendran, Chung‐Li Dong, Dong Jin Yoo

**Affiliations:** ^1^ Department of Energy Storage/Conversion Engineering of Graduate School (BK21 FOUR), Hydrogen and Fuel Cell Research Center Jeonbuk National University Jeonju‐si Jeollabuk‐do 54896 Republic of Korea; ^2^ Research Center for X‐Ray Science Department of Physics Tamkang University Tamsui 25137 Taiwan; ^3^ Department of Chemistry School of Science and Humanities Vignan's Foundation for Science, Technology and Research Vadlamudi Guntur Andhra Pradesh 522213 India; ^4^ Green Energy & Applied Research (GEAR) Lab, Department of Environmental Science and Engineering SRM University AP Amaravati 522240 Andhra Pradesh India; ^5^ Department of Life Science Jeonbuk National University Jeonju‐si Jeollabuk‐do 54896 Republic of Korea

**Keywords:** Fe—C interactions, iron nanoparticles, porous bio‐carbon materials, redox reactions, Zn–air batteries

## Abstract

In practical applications, zinc–air batteries (ZABs) require high‐performance, durable, and cost‐effective electrocatalysts for the critical oxygen reduction reaction (ORR) and oxygen evolution reaction (OER). Here, we describe a reflux synthesis method of constructing a porous catalyst by introducing turmeric yellow into extremely porous bio‐carbon (PC) materials that contain iron nanoparticles (Fe NPs); these catalysts are known as Fe NPs@PC. These catalysts have become a significant substitute for high‐performance cathodes in ZABs because their electrochemical properties can improve ORR performance. In addition to enhancing conductivity, the OER/ORR bifunctional active sites must be balanced by optimizing the Fe—C and Fe—Fe interactions within the active site. X‐ray absorption analysis and density functional theory confirmed that strong iron‐carbon interactions promote OER (*η*_10_ = 320 mV) and ORR (*E*_1/2_ = 0.786 V) activity and exhibit a smaller potential gap of 0.764 V of Fe NPs@PC‐700 catalyst. The impact of this redox activity enhances the high‐power density (219 mW cm^−2^) and long‐term charge–discharge cycle stability (85 h@3 mA cm^−2^) of ZABs. This work charts a viable route for the assembly of practical ZABs by regulating bifunctional electrocatalysts via appropriate modification of active sites.

## Graphical abstract

The synthesis of environmentally benign bio‐carbon‐supported metal nanoparticles and a brief study of the iron–carbon interaction enhance the redox activity of oxygen evolution reaction and oxygen evolution reaction techniques in Zn–air batteries.

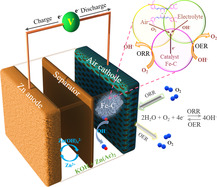

## 1 Introduction

The demand for safe, reasonably priced, and ecologically friendly energy‐generating and storage solutions is growing as the size and technological impact of civilization advance at a rapid pace.^[^ ^1^, ^2^, ^3^ ^]^ Because of their low cost, sustainability, and high theoretical energy density (1218 W h kg^−1^), rechargeable zinc–air batteries (ZABs) are among the most promising candidates for the next generation of energy‐storage devices.^[^ ^4^, ^5^, ^6^ ^]^ ZABs are rechargeable energy systems that need an air cathode to catalyze both the oxygen evolution reaction (OER), which corresponds to the charging process, and the oxygen reduction reaction (ORR), the discharging process.^[^ ^7^, ^8^, ^9^ ^]^ However, because the OER and ORR are kinetically sluggish, large charge/discharge voltage variations and low energy efficiency are common obstacles to commercial deployment.^[^ ^10^, ^11^ ^]^

Precious metals such as platinum, iridium, and ruthenium are typically used to achieve effective ORR and OER.^[^ ^12^, ^13^ ^]^ However, high costs and the need for greater durability and bifunctional qualities limit the use of precious metals.^[^ ^14^, ^15^ ^]^ Thus, it is essential to identify economical, recyclable, and efficient redox‐activity catalysts. Prior research has focused on alkaline aqueous electrolytes with a zinc anode and a porous carbon‐based air cathode.^[^ ^8^, ^16^ ^]^ Because of their electrical conductivity, stability, customizable shapes, and practicality, carbon compounds are attractive materials for electrodes in commercial ZABs.^[^ ^17^, ^18^ ^]^ As air electrodes for ZABs, a variety of carbon materials, including Super P,^[^ ^19^ ^]^ graphene,^[^ ^20^ ^]^ and carbon nanotubes,^[^ ^21^ ^]^ have demonstrated outstanding electrical conductivity and programable topologies. Unfortunately, market application of these materials as electrode materials has been limited due to the cost of their raw materials, complex preparation techniques, need for large amounts of energy, and pollution.^[^ ^18^, ^22^ ^]^ Turmeric yellow (diferuloylmethane) is a biomass chemical that is readily available,^[^ ^23^, ^24^, ^25^ ^]^ which may be a viable source of active catalytic carbon materials for the production of redox electrocatalysts in air electrodes or for direct use as electrode materials.^[^ ^26^, ^27^ ^]^ Earth‐abundant active metals are another possibility to further improve catalytic endurance with the use of doped carbon materials.

Abundant iron‐based materials, including nanoparticles (Fe NPs), Fe_3_C, single‐atom catalysts, Fe_5_C_2_, and Fe_2_O_3_, have been shown to be alternative ORR electrocatalysts among non‐noble‐metal catalysts.^[^ ^28^ ^]^ Iron NPs frequently exhibit attractive electrocatalytic performances when combined with conductive substrates such as graphene and biomass.^[^ ^29^ ^]^ Recent years have seen an increase in interest in Fe NPs as effective ORR electrocatalysts due to their numerous active sites, mass activity, and atom‐utilization efficiency approaching 100%.^[^ ^30^, ^31^ ^]^ However, their simple geometries and coordination structures, catalytic activity, and stability hinder practical use.^[^ ^32^ ^]^ For sophisticated Fe‐based catalysts, substrates with complex structures must be designed and controlled.

To demonstrate the reflux and pyrolysis processes involved in NPs that combine Fe with highly porous bio‐carbon (Fe NPs@PC with different temperatures at 600 °C, 700 °C, and 800 °C), we used turmeric yellow as a carbon source. The combination produced a highly porous structure with interacting Fe NPs that permit a highly effective redox activity. This approach to the fabrication of an Fe NPs@PC catalyst offers the possibility as a sustainable redox electrocatalyst for use on a large scale with minimal pollution and at relatively low cost. Transmission electron microscopy (TEM), X‐ray absorption spectroscopy (XAS), and density functional theory (DFT) were used to analyze the prepared catalyst. The results showed uniformly developed particles and a measurable interaction between Fe and C. The Fe NPs engaged with active sites on the carbon through electrochemical processes that involve electron redistribution and increased NP stability, augmenting the catalytic activity associated with the ORR and OER.^[^ ^28^, ^33^, ^34^ ^]^ The catalytic behavior of Fe NPs@PCs toward both the OER and ORR is more promising than that of any bifunctional bio‐carbon‐derived electrocatalysts reported. The Fe NPs@PC‐700 catalyst was used to produce a rechargeable ZAB with a specific capacity of 754 mAh g^−1^_Zn_ in atmospheric conditions and a discharge power density of 219 mW cm^−2^ using Fe NPs@PC‐700 for air electrodes. The Fe NPs@PC catalyst offers superior rechargeability and long‐term stability in ZABs.

## 2 Results and Discussion

**Figure 1:**
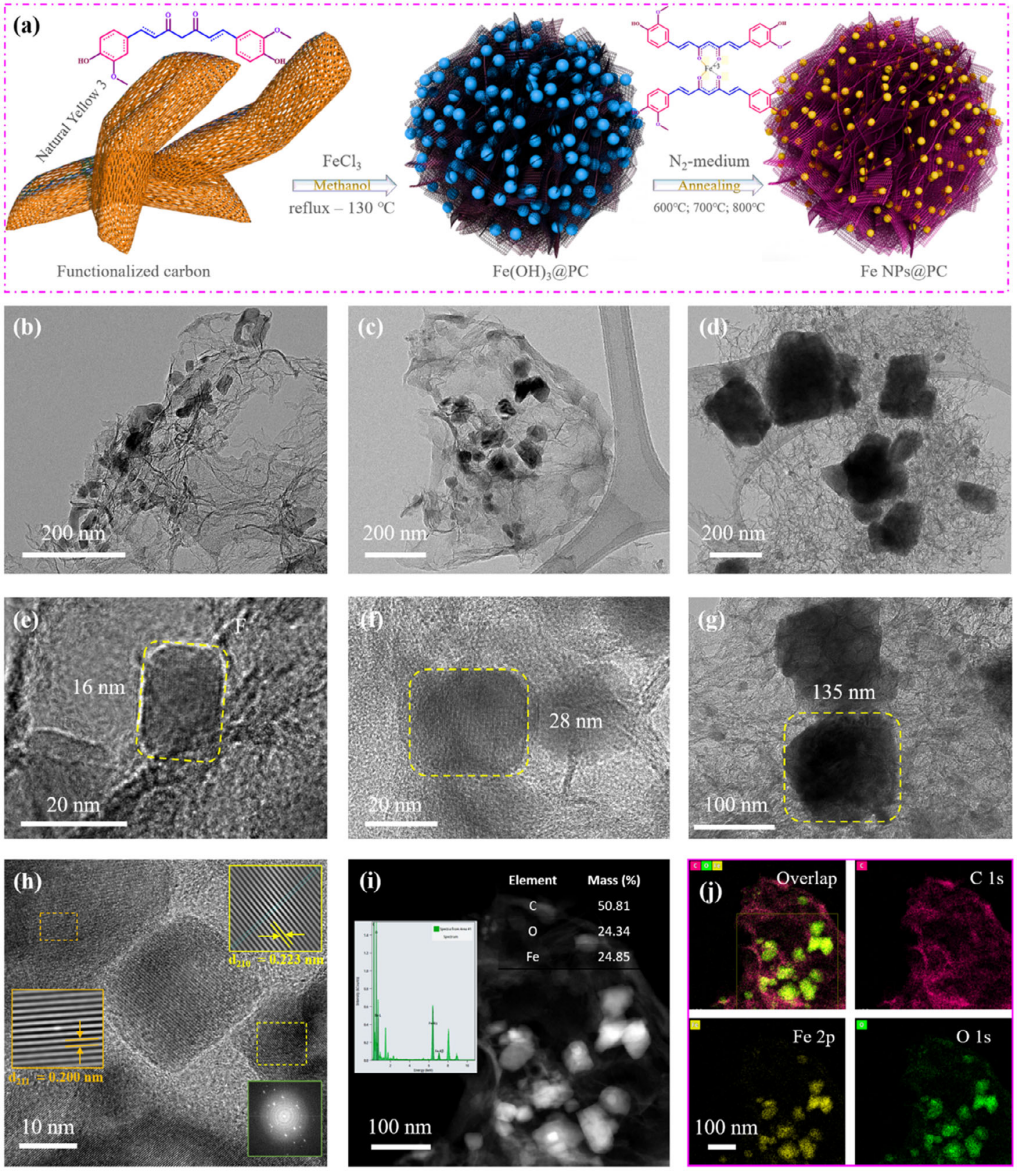
a) A schematic of the synthesis of Fe nanoparticles on a carbon composite. b–d) TEM images and e–g) HR‐TEM images of Fe NPs@PC‐600, Fe NPs@PC‐700, and Fe NPs@PC‐800 catalysts. h) HR‐TEM, i,j) TEM‐EDS elemental mapping, and TEM‐EDX spectrum of the Fe NPs@PC‐700 catalyst.

Fe NPs supported on highly porous bio‐carbon were produced using the reflux technique to maintain the reaction at a steady temperature without loss of solvent.^[^ ^35^ ^]^ As seen in **Figure** **1**a, the turmeric yellow was kept in a methanol solution. Iron (III) chloride hexahydrate (FeCl_3_. 6H_2_O) and a urea solution were then added dropwise while being stirred at room temperature. Eventually, a small amount of ammonium fluoride was introduced as a structure‐tuned reagent to create the metal hydroxide with a bio‐carbon combination (Equation (8)–(11). At 130 °C, the solution continued to spin and reflux even after being washed several times with ethanol and DI water. The powder was pyrolyzed in an inert environment for 2 h at different temperatures for 600 °C, 700 °C, and 800 °C, which are referred to as Fe NPs@PC‐600, Fe NPs@PC‐700, and Fe NPs@PC‐800. Methanol and ammonium fluoride are essential to this synthesis technique as both assist in the production of a porous carbon framework with Fe NPs in the porous regions.^[^ ^36^, ^37^ ^]^ As a solvent, methanol aids in the synthesis of an intermediate substance, like a metal–organic framework nanocomposite, which in turn develops into the precursor for porous carbon. Ammonium fluoride has a tendency to influence the final porous carbons or the intermediate compounds' arrangement. Finally, characterization techniques were used to establish the production of Fe NPs@PC catalyst.

The SEM images in Figure S1a–f, Supporting Information, demonstrate the growth of highly porous carbon. The formation of porous carbon was preserved when appropriate amounts of carbon atoms were present, and the carbon porous diameters increased gradually from 300 to 500 nm. Porous materials improve electron transfer and increase mass transport compared to less porous materials. Furthermore, Fe atoms with nanoparticle structures were introduced to the extremely porous carbon framework to generate Fe NPs, which occupy the porous position for particles with a diameter of ≈125 nm. Figure S2a,d,g, Supporting Information, illustrates the porous structure of the inner layer of the Fe NPs@PC‐600, Fe NPs@PC‐700, and Fe NPs@PC‐800 catalysts and the associated highly porous carbon and Fe NPs.

Figure S2b,e,h, Supporting Information, shows the combined accurate atomic percentages of Fe and C as revealed by high‐angle annular dark‐field imaging combined with EDS projections. Also, further confirm the synthesis of an Fe NPs@PC‐600, Fe NPs@PC‐700, and Fe NPs@PC‐800 catalyst by displaying the elemental mapping of the iron and carbon atoms with the EDX spectrum in Figure S2c,f,i, Supporting Information. A small percentage of oxygen atoms is visible in the EDX spectrum, indicating the existence of a surface oxidant in the Fe NPs@PC catalysts, but it is not linked to the construction of structures.

Using atomic force microscopy (AFM), the surface roughness of the Fe NPs@PC‐600, Fe NPs@PC‐700, and Fe NPs@PC‐800 catalyst was further examined; the findings are displayed in Figure S3a–c, Supporting Information. The AFM images of the Fe NPs@PC catalyst demonstrate that the Fe NPs were anchored on highly porous bio‐carbon, and three‐dimensional AFM images of the catalyst reveal that the calculated surface roughness was ≈200 nm. This strengthened the electrochemical energy conversion applications by improving the absorption of analytes of target molecules on the surface of the Fe NPs@PC catalyst. The N_2_ adsorption parameters used to measure the surface area and porous properties of the Fe NPs@PC‐600 catalyst in a Brunauer–Emmett–Teller analysis are shown in Figure S4, Supporting Information.^[^ ^38^, ^39^ ^]^ This analysis confirmed creations of a highly porous carbon with metal particle catalyst at the ideal annealing temperature of 600 °C; the optimized Fe NPs@PC‐600 had an improved specific surface of 34.89 m^2^ g^−1^. Additionally, Barret–Joyner–Halenda modeling revealed fluctuations in the pore size of the Fe NPs@PC‐600.^[^ ^40^, ^41^ ^]^ The Fe NPs@PC was mesoporous, as shown by the insert in Figure S4, Supporting Information, as evidenced by the pore diameters of 23.96 nm. Together with their unique mesoporous nature, the large pore diameters and high specific surface area of the Fe NPs@PC‐600 can improve transportation kinetics and increase the number of active sites for energy storage and conversion. Since the Fe NPs@PC composites' entire structure has robust metal binding and greater intrinsic activity.

The Raman spectra of the bio‐carbon and Fe NPs@PC‐600 composites shown in Figure S5a, Supporting Information, further validate the structural conversion that occurred during the synthesis process. In the porous carbon and Fe NPs@PC composites, the primary peaks at 1334.2 and 1579.5 cm^−1^ correspond to defective (D) and graphitic (G) bands.^[^ ^42^ ^]^ Consequently, the calculated *I*_D_/*I*_G_ ratio for the produced material was 1.08 for porous carbon. The Fe NPs@PC‐600 (0.94), Fe NPs@PC‐700 (0.97), and Fe NPs@PC‐800 (1.02) composites exhibited notable G and D bands upon addition of Fe NPs, demonstrating the breakdown of the hybrid metal–organic architecture. The Raman spectra show characteristic peaks that match the *E*_g_ modes in Fe NPs as well as other distinct Fe—C peaks.^[^ ^43^ ^]^ The vibrational band of the resulting Fe NPs peak was observed at 372.4 cm^−1^. The magnitude of these two bands was changed by addition of Fe NPs, which fix metallic iron onto porous carbon composites. Additionally, a higher defect count enhances the electrocatalytic efficiency and reaction kinetics of carbon‐based materials.

Fourier‐transform infrared (FT‐IR) analysis was used to examine the biological functionalization of the porous carbon, Fe NPs@PC‐600, Fe NPs@PC‐700, and Fe NPs@PC‐800 composite and bio‐carbon, as illustrated in Figure S5b, Supporting Information. According to the bio‐carbon FT‐IR spectra, the peaks at 3456, 2921, 2852, and 1632 cm^−1^ correspond to hydroxyl O—H stretching, —CH_2_‐bridges, aromatic C—H stretching vibrations, and C=O (epoxide group) stretching, respectively.^[^ ^44^, ^45^ ^]^ For the highly porous bio‐carbon, the peak for C—H bending modes was at 1090 cm^−1^. The remaining absorption peaks were the same as for porous bio‐carbon, with additional peaks appearing at 1007.3 and 686.2 cm^−1^ corresponding to the respective Fe—C and Fe—O bonds of the Fe NPs@C composite.^[^ ^46^ ^]^ These FT‐IR data clearly show that the chemical reflux approach creates identical porous carbon and Fe NPs@PC composites.

The analysis of structural characteristics using high‐resolution images of highly permeable bio‐carbon‐assisted Fe NPs@PC‐600, Fe NPs@PC‐700, and Fe NPs@PC‐800 based on TEM analysis. A cube‐like structure with extremely porous bio‐carbon incorporating Fe NPs evenly dispersed throughout the structure is depicted in Figure 1b–d. Because of the porous structure, methanol was used to determine the duration of reaction. Additionally, the cube size was verified using Gatan microscopy software to measure its different temperatures annealing increase the distances, which were 16, 28, and 135 nm, respectively (Figure 1e–g). ImageJ software was used to calculate the types of nanoparticles. The high magnification in the TEM image of porous carbon (Figure S6a,b and S7a,b, Supporting Information) displays a particle‐shaped structure that contains Fe NPs in Fe NPs@PC‐600 and Fe NPs@PC‐800. Mainly, Fe NPs@PC‐700 show that the particles were ≈28 nm in size for evenly dispersed porous carbon. The high‐resolution TEM photograph in Figure 1h features two types of particles with crystalline structures: 211 is the 0.20 nm, and 210 is the 0.223 nm.

**Figure 2:**
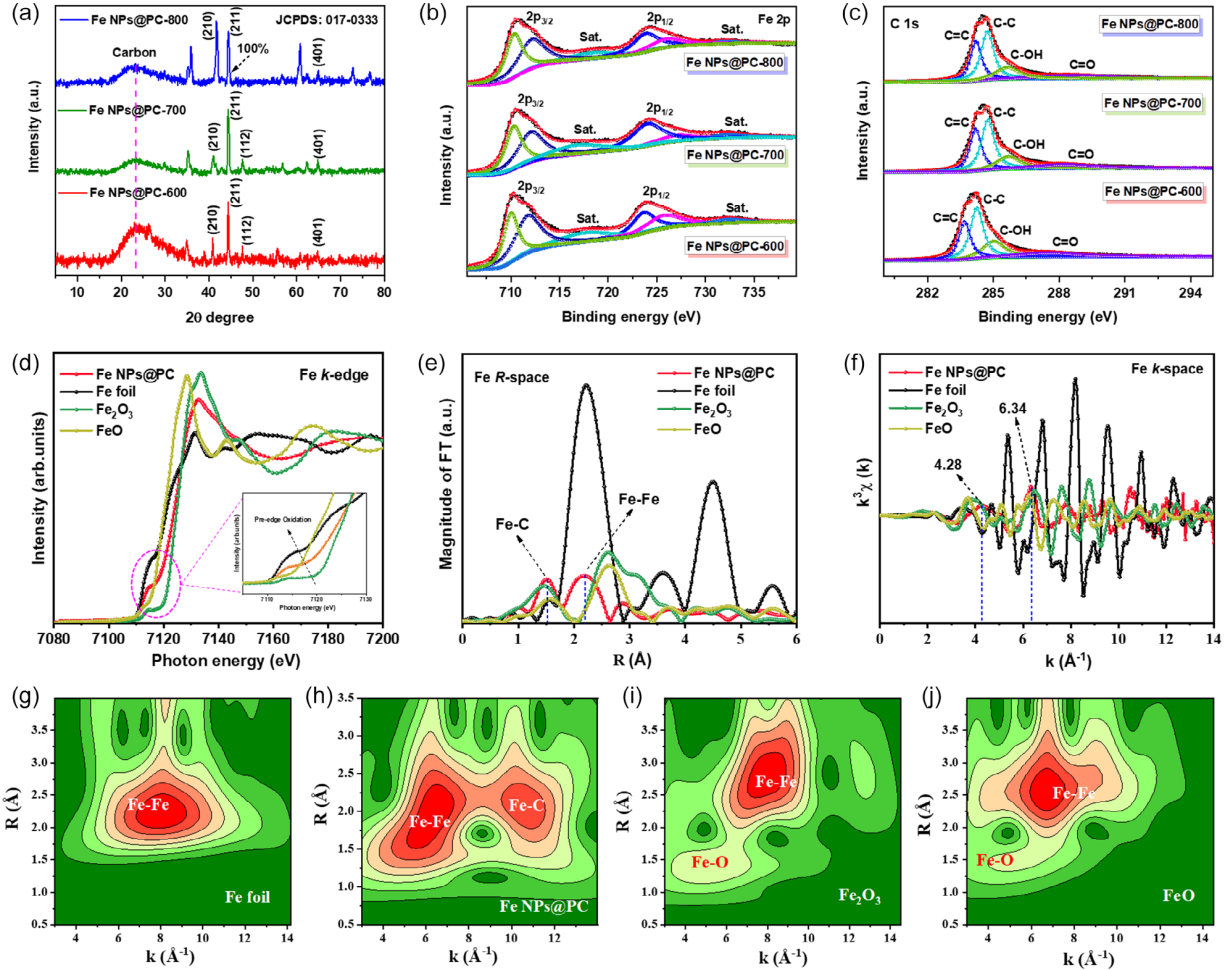
a) XRD analysis of Fe NPs@PC‐600, Fe NPs@PC‐700, and Fe NPs@PC‐800 catalysts. High‐resolution XPS spectra of (b) Fe 2p and (c) C 1s Fe NPs@PC‐600, Fe NPs@PC‐700, and Fe NPs@PC‐800 composites. XAS analysis of Fe k‐edge (d) XANES spectrum, (e) EXAFS spectrum, and (f) EXAFS k‐space spectrum, and (g–j) wavelet transforms that correlate with a k_3_‐weighted Fe K‐edge spectra of Fe foil, Fe NPs@PC, commercial Fe_2_O_3_, and FeO catalysts, respectively.

For the Fe and C nanoparticles in Figure 1h, high‐resolution TEM images and fast Fourier‐transform patterns verify the existence of a matching crystalline phase (211 and 210). Furthermore, the crystalline line spectra that matched the appropriate phase line are aligned. Following that, we determined which crystal lines in **Figure** **2**a were associated with the appropriate XRD planes. Figures S8, Supporting Information, present one of the most significant fragments of evidence supporting particle production in both dark‐field and bright‐field TEM images.

High‐angle annular dark‐field (HAADF) imaging with atomic resolution combined with spherical aberration–corrected scanning TEM allowed differentiation of these two types of particles (Figure 1i,j). The detection of Fe NP carbon clusters or particles is indicated by the diffuse bright spots in the HAADF images, confirming that the Fe NPs diffused uniformly in highly porous bioinspired carbon in Fe NPs@PC‐700. The presence of Fe and C atoms in the catalyst was revealed by EDS color imaging of Fe NPs@PCs at a comparatively low beam current (Figure 1i,j). The elemental percentages and EDX spectra verified the levels of Fe (24%) and C (50%) present (Figure 1j). The transparent EDX spectra and EDX mapping state of the Fe NPs@PC‐600 and Fe NPs@PC‐800 catalysts were verified by TEM, which also revealed the porous structure. This unique structure can accelerate electron transfer and kinetic transfers in the Fe NPs@PC‐600 and Fe NPs@PC‐800 catalyst, allowing it to efficiently store energy and power conversion devices (Figure S6c,d and S7c,d, Supporting Information).

As seen in Figure S8, Supporting Information, XRD analysis was used to reveal the phase and crystallinity of the bio‐carbon composites, producing an XRD peak for carbon that corresponds to the (002) and (101) planes at 23.0° and 43.3°, respectively. The addition of an Fe atom activates epoxy and carboxyl bonds on the bio‐carbon composite with a matching d‐spacing of 0.32 nm (002), which shows in Figure 2a. After the addition of the Fe atom, the diffraction peaks at 40.8°, 44.4°, 47.5°, and 64.7° in the Fe NPs@PC‐600, Fe NPs@PC‐700, and Fe NPs@PC‐800 composite XRD patterns correspond to the (210), (211), (112), and (401) crystal planes, respectively.^[^ ^46^ ^]^ These findings are in agreement with a typical scattering pattern (JCPDS 017–0333). We used the Scherrer formula to calculate a crystallite size distribution of Fe NPs as ≈0.20 nm based on a distinctive peak of 44.4°. These findings show that the extremely porous bio‐carbon surface was coated with homogeneous, highly crystalline Fe NPs. The Vienna Ab initio Simulation Package was used to simulate the two types of crystal structures for simple cubic Fe and Fe_7_C_3_ based on XRD planes, as shown in Figure S9a,b, Supporting Information.

We also used XPS to analyze the chemical structure and oxidation states of biomass carbon (Figure S10, Supporting Information), Fe NPs@PC‐600, Fe NPs@PC‐700, and Fe NPs@PC‐800,^[^ ^2^ ^]^ which demonstrates (Figure S11a,b, Supporting Information) the presence of C 1s, O1s, and Fe 2p. Two major peaks representing Fe 2p_3/2_ and Fe 2p_1/2_ were seen in the deconvoluted spectra of the Fe 2p region of the spectrum in Fe NPs@PC at 712.1 and 725.8 eV, respectively. Additionally, deconvoluting the Fe 2p_3/2_ peak revealed two peaks corresponding to Fe^3+^ and Fe^2+^ at 710.1 and 713.5 eV, respectively.^[^ ^47^ ^]^ Similarly, two peaks at 723.3 and 728.1 eV in the deconvoluted Fe 2p_1/2_ peaks were associated with Fe^3+^ and Fe^2+^, respectively, and were part of the Fe NPs@PC‐600, Fe NPs@PC‐700, and Fe NPs@PC‐800 composites. Furthermore, Fe 2p_3/2_ and Fe 2p_1/2_ were represented by two satellite peaks at 718.2 and 734.9 eV, respectively (Figure 2b). Figure 2c depicts the C 1s spectra of the deconvoluted porous bio‐carbon and Fe NPs@PC composites. The peaks at 284.1, 284.5, 285.9, and 288.3 eV can be attributed to C=C, C—O, C=O, and O—C=O, respectively.^[^ ^3^ ^]^ Furthermore, there is a discernible change in the C 1s spectra C=O and O—C=O, confirming the presence of Fe NPs in the bio‐carbon platform and providing solid evidence for the creation of Fe NPs@PC‐600, Fe NPs@PC‐700, and Fe NPs@PC‐800 composites. Figure S11c, Supporting Information, displays the O 1s high‐resolution deconvoluted spectra, which have peaks at 530.8, 531.7, 532.4, and 533.3 eV. The C—O—Fe species in Fe NPs@PC, which belong to residual oxygen‐containing groups, are responsible for the peak at 531.5 eV.

Following confirmation of the oxidation states and spin splitting for the Fe 2p spectrum using XPS analysis, the absorbing atom's oxidation state, coordination geometry, and local electronic environment were examined using XAS analysis.^[^ ^48^, ^49^ ^]^ Further X‐ray absorption near‐edge structure (XANES) studies and extended X‐ray absorption fine structure (EXAFS) spectra were obtained to characterize the atomic structure of Fe NPs@PC. The Fe K‐edge XANES spectrum in Figure 2d indicates that the pre‐edge location of Fe NPs@PC is related to Fe foil, FeO, and Fe_2_O_3_, indicating that the overall state of oxidation of the Fe atoms in Fe NPs@PC is probably an Fe valence state of +3. The pre‐edge peak confirmed oxidation of the Fe atom with a peak shift at 7110.8 eV, which indicates faster oxidation of the Fe NPs@PC catalyst compared with other reference materials (inset, Figure 2d). One of the most compelling pieces of evidence for Fe—C creation without Fe—O formation is the lower intensity of absorption (7130.9 eV) of the postedge peaks of the Fe NPs@PC catalyst compared with those of Fe_2_O_3_ and FeO. The Fe‐inexpensive and plentiful NPs@PC composite catalyst allowed electrochemical interaction of Fe and C atoms.

**Figure 3:**
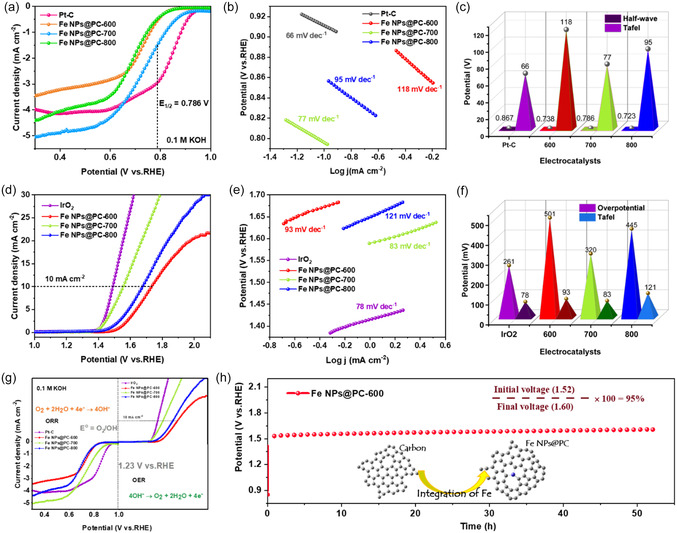
An ORR study in a 0.1 M KOH electrolyte solution: (a) LSV curves at rotation speeds of 1600 rpm, (b) Tafel slopes, and (c) a bar‐chart diagram of half‐wave potential and Tafel slopes of commercial Pt‐C and Fe NPs@C‐600, Fe NPs@PC‐700, and Fe NPs@PC‐800 electrocatalysts. Results of an OER study in a 0.1 M KOH electrolyte solution: (d) LSV curves at a scan rate of 10 mV, (e) Tafel slopes, (f) comparison of overpotential and Tafel values. (g) Oxygen electrocatalyst activities in the range of potential in the OER and ORR (O_2_‐saturated at 1600 rpm). (h) Chronopotentiometry long‐term stability at 10 mA current density for Fe NPs@PC‐600 electrocatalyst.

A Fourier‐transformed EXAFS analysis of the Fe NPs@PC for the R‐space spectrum of Fe is displayed in Figure 2e. With no indications of the Fe—O coordination space, the peak at 1.45 Å can be attributed to the interaction of Fe NPs with carbon atoms (Fe—C coordination).^[^ ^25^, ^50^ ^]^ Because of their strong interactions with oxygen atoms, Fe_3_O_4_, FeO, and Fe foil have stronger oxidation peaks. Additionally, Fe—Fe coordination is seen at 2.2 Å of Fe NPs@PC and Fe_2_O_3_, while that of FeO is shown at 2.60 Å. This significant reduction in R‐space value indicates robust growth with Fe—C coordination environment without Fe—O bond associations in Fe NPs@PC catalyst. The atomic structure of Fe atoms plays a crucial role in spin‐splitting states, as seen in Figure S9c, Supporting Information. The k_3_‐weighted EXAFS spectrum further confirmed the coordination states by displaying tetrahedral and octahedral geometries at 4.28 and 6.34 Å^−1^ (Figure 2f). In fact, both Fe k_3_‐weighted EXAFS (**Figure** **3**e) and wavelet transforms (Figure 2g–j) confirm the formation of Fe—C and Fe—Fe bonds in Fe NPs@PC‐600, while Fe—O and Fe—Fe bonds in Fe_2_O_3_, FeO, and Fe foil, respectively. The above observation further demonstrates the successful formation of Fe–C and Fe–Fe coordination in Fe NPs@PC‐600 catalysts (see Figure S12a–c, Supporting Information), and indicates a typical charge‐transfer efficiency between Fe NPs@C that results in improved conductivity and effective reaction kinetics.

## 3 Electrochemical Performance of the OER and ORR Using Fe NPs@PC Catalysts

The OER and ORR are essential processes in electrochemistry and renewable energy technologies, including metal‐air batteries and fuel cells.^[^ ^51^ ^]^ Despite sluggish kinetics and large overpotentials, the ORR involves the reduction of oxygen, and the OER involves the evolution of oxygen. For electrochemical energy systems such as metal–air batteries, the ORR is essential. For electrochemical energy systems such as metal–air batteries, the OER is essential. In applications involving electrochemical renewable energy, both the OER and ORR are crucial.^[^ ^52^, ^53^ ^]^

(1) $$O_{2}{+\;4H}^{+}{+\;4e}^{-}\to {\text{ 2H}}_{2}\text{O }\left(\text{ORR}\right)$$

(2) $${\text{2H}}_{2}\text{O }\to {\text{ 4e}}^{-}{\text{+ 4H}}^{+}{\text{+ O}}_{2}\left(\text{OER}\right)$$

Several electrochemical techniques were used to assess the catalysts electrochemical properties, geometry, composition, and structural features. Its dual‐functional oxygen electrocatalytic activity was investigated by analyzing its ORR performance for using Fe NPs@PC‐600, Fe NPs@PC‐700, and Fe NPs@PC‐800 electrocatalysts. Cyclic voltammograms (CVs) in 0.1 M KOH, concentrated with N_2_ and O_2_, were used to examine the electrocatalytic activity of the catalysts produced for the ORR. As shown in Figure S13, Supporting Information, the catalysts in the N_2_‐saturated state did not exhibit a reduction peak. In contrast, distinct cathodic reduction peaks emerged once an electrolyte was saturated with O_2_. The intensity and location of these reduction peaks determine the ORR electrocatalytic activity. Fe NPs@PC‐700 achieved superior ORR performance compared with each of the reported bio‐carbon–based catalysts except Pt/C (≈0.867 V), with an improved positive peak potential at approximately ≈0.786 V.

A rotating disk electrode (RDE) was used to obtain linear sweep voltammograms (LSVs) of the various catalysts at 1600 rpm in a KOH solution saturated with 0.1 M O_2_ (Figure 3a). Unless otherwise indicated, a blank response captured under N_2_ was used to adjust the current density. Fe NPs@PC‐700 had marginally superior results (*E*_onset_ = 0.917 V, E_1/2_ = 0.786 V), primarily because single nanoparticles for effective ORR create a stable state that results in a large number of active sites (Table S1, Supporting Information). Compared with the other synthesis materials (Fe NPs@PC‐600; *E*_onset_ = 0.849 V, E_1/2_ = 0.733 V, and Fe NPs@PC‐800; *E*_onset_ = 0.845 V, E_1/2_ = 0.715 V), Fe NPs@PC‐700 showed superior values similar to those of commercial Pt/C (*E*_onset_ = 0.95 V and *E*_1/2_  = 0.87 V). Moreover, Figure 3a demonstrated that the limit current for Fe NPs@PC‐700 (5.0 mA cm^−2^) had faster ORR kinetics compared with that of Pt/C (4.16 mA cm^−2^). Next, a created material's Tafel slope (Figure 3b) was computed for assessing rate kinetics and identifying the mechanistic pathway of the electrochemical reaction.^[^ ^54^ ^]^ The reaction kinetics at Fe NPs@PC‐700 appeared to be rapid, as evidenced by the significantly smaller Tafel slope for Fe NPs@PC‐700 (77 mV dec^−1^) than for Pt/C (66 mV dec^−1^), as lowered with Fe NPs@PC‐600 (118 mV dec^−1^) and Fe NPs@PC‐800 (95 mV dec^−1^).

The LSVs obtained from different rotation speeds and related computations were used to examine the electron‐transfer process through the ORR with different rotation speeds of 400–2800 rpm (Figure S14a–d, Supporting Information). Figure S15a–c, Supporting Information, depicts the Koutecky–Levich (K–L) relationship, which shows that the current density increased with rotation speed for an Fe NPs@PC‐600, Fe NPs@PC‐700, and Fe NPs@PC‐800 altered RDE within a potential range of 0.3–0.6 V. With nearly equal slopes and strong linear relationships, all K–L plots were equivalent to those of commercial Pt/C, indicating that the ORR proceeded according to first‐order kinetics.^[^ ^55^, ^56^ ^]^ A 4e^−^ ORR mechanism was confirmed by the K–L equation, which yielded an electron‐transfer number of 3.28 for the ORR of Fe NPs@PC‐700. The exact process by which H_2_O_2_ produced in one location can be transformed into water at another has yet to be identified.^[^ ^57^ ^]^ Atomic splitting (2H_2_O_2_ → O_2_ + 2H_2_O) or electroreduction (H_2_O_2_ + 2 H^+^ + 2e → 2H_2_O) may accomplish this process. The O_2_ generated during the reaction of disproportionation can be reduced to H_2_O_2_ in the latter case. Repeating such cycles on a sizable number of O_2_ molecules would result in an apparent 4e^−^ decrease of O_2_, even in the improbable case that H_2_O_2_ is barely electroreduced to H_2_O.^[^ ^58^, ^59^ ^]^ Figure 3c illustrates this difference, using prepared electrocatalysts that applied half‐wave potential and Tafel values, linking it to the disintegration and accumulation of precious atoms during this time (Table S1, Supporting Information).

An OER analysis involving three types of electrodes (a platinum rod as counter, Ag/AgCl as reference, and Fe NPs@PC‐600, Fe NPs@PC‐700, and Fe NPs@PC‐800 working electrodes, respectively) demonstrated outstanding ORR activity.^[^ ^60^ ^]^ When using a 0.1 M KOH solution at a scan rate of 25 mV s^−1^, the resulting CV curves for Fe NPs@PC‐600, Fe NPs@PC‐700, Fe NPs@PC‐800, and IrO_2_ (Figure S16, Supporting Information) show enhanced oxidation and reduction peaks. The OER efficiency of the Fe NPs@PC‐700 catalyst was relatively high (Figure 3d), as evidenced by its lower overpotential in the LSV curve (*E*_η10_ = 1.550 V), which was near than that of the IrO_2_ catalyst (*E*_η10_ = 1.495 V) and lower than that of Fe NPs@PC‐600 (*E*_η10_ = 1.731 V) and Fe NPs@PC‐800 (*E*_η10_ = 1.675 V), other bio‐carbon with iron catalysts, which were comparable. Table S1, Supporting Information, lists recent research on Fe catalysts, and Figure S20b, Supporting Information, compares overpotentials of 10 cm^−2^ for Fe NPs@PC‐600, Fe NPs@PC‐700, Fe NPs@PC‐800, and IrO_2_. This clearly demonstrates that the addition of Fe NPs to Fe NPs@PC‐700 electrocatalysts causes them to become more active than other Fe electrocatalysts that incorporate bio‐carbon.

In contrast to IrO_2_, which has a Tafel slope of 78 mV dec^−1^, Fe NPs@PC‐600, Fe NPs@PC‐700, and Fe NPs@PC‐800 have a Tafel slope of 93, 83, and 121 mV dec^−1^, as shown in Figure 3e. The Tafel slope, which is typically used to examine OER electrocatalysis, is evidence of the faster reaction kinetics of Fe NPs@PC‐700 (83 mV dec^−1^) electrocatalysts toward OER. Figure 3f compares the OER performance of Fe NPS@PC‐600, Fe NPS@PC‐700, Fe NPS@PC‐800, and IrO_2_ electrocatalysts, demonstrating the more affordable and readily available electrocatalyst of Fe atoms associated with bio‐carbons that successfully activate electrochemical performance. According to electrochemical impedance spectroscopy (EIS), the produced catalysts exhibited a modest charge‐transfer resistance (*R*_ct_), as demonstrated by the values of Fe NPs@PC‐600 (8.19 Ω), Fe NPS@PC‐700 (1.03 Ω), Fe NPS@PC‐800 (3.0 Ω), and IrO_2_ (1.72 Ω) in Figure S17a,b, Supporting Information.^[^ ^61^, ^62^ ^]^ An equivalent electrical circuit (insert in Figure S17a), which depicts the electrochemical reactions taking place at the different electrode/electrolyte interfaces, was used to match the EIS data for interpretation. The series resistance (*R*_s_), charge‐transfer resistance (*R*_1_ and *R*_2_), and capacitance of the constant‐phase element (*Q*_1_ and *Q*_2_) of the counter electrode and working electrode were all measured. As charges were transported to the collecting electrode, the *R*_s_ primarily came from the contact between the electrocatalytic material and the current‐receiving electrode (carbon paper).^[^ ^63^, ^64^ ^]^ Electrolyzing the species at their interface and indicating a typical charge‐transfer efficiency between Fe NPs@PC revealed improved conductivity and effective reaction kinetics.

The source of increased OER activity was evaluated using the electrochemically active surface area (ECSA) derived from CV curves obtained at various scan speeds in the non‐Faradaic zone to measure the charged double‐layer capacitance (*C*_dl_) of the generated electrocatalysts.^[^ ^21^, ^65^ ^]^ As shown in Figures S18a–d and S18e, Supporting Information, the *C*_dl_ values of porous Fe NPs@PC‐600, Fe NPs@PC‐700, Fe NPs@PC‐800 and IrO_2_ were 1.8, 0.91, 0.246, and 0.77 mF cm^−2^, respectively. Among all the materials analyzed, it was determined to have the highest ECSA and the most active sites, which led to the best OER performance of IrO_2_ (19.5 cm^2^) and Fe NPs@PC‐600 (45 cm^2^), Fe NPs@PC‐700 (22.75 cm^2^), Fe NPs@PC‐800 (6.15 cm^2^) (Figure S18f, Supporting Information). Figure 3h also displays the results of a chronopotentiometry durability test of the Fe NPs@PC‐600 electrocatalyst at a charge density of 10 mA for 50 h. According to the results, the Fe NPs@PC‐600 electrocatalyst demonstrated 95% steady‐state endurance for the estimated initial versus final voltage, maintaining a stable potential for more than 30 h. These findings are in contrast to those of newly released studies on LSV overpotential that use porous bio‐carbon‐supported, Fe‐based electrocatalysts (Figure S20b, Supporting Information).

The *E* value (Δ*E* = *E*_j=10_OER − *E*_1/2_ORR) was used to determine the Fe NPs@PC electrocatalyst OER/ORR dual‐functional behavior, which was then compared to the characteristics of the other materials. In general, there was an inverse relationship between Δ*E* and the bifunctional oxygen electrocatalyst efficiency.^[^ ^2^, ^66^ ^]^ Fe NPs@PC‐700 had the lowest *E* value at 0.764 V compared with 0.625 V for the Pt/C + IrO_2_ electrocatalyst (Figure 3g) according to the overall ORR/OER polarization curves of the catalysts (Table S1, Supporting Information) except for Pt/C + IrO_2_ (Fe NPs@PC‐600; 0.993 V and Fe NPs@PC‐800; 0.952 V). A DFT analysis of the carbon generated from Fe‐doped turmeric yellow was carried out to investigate the effect of the activating metal on the dual‐functional properties of the Fe NPs@PC electrocatalyst.

## 4 Performance of Zn–Air Battery Application with Fe NPs@PC Air Electrode

**Figure 4:**
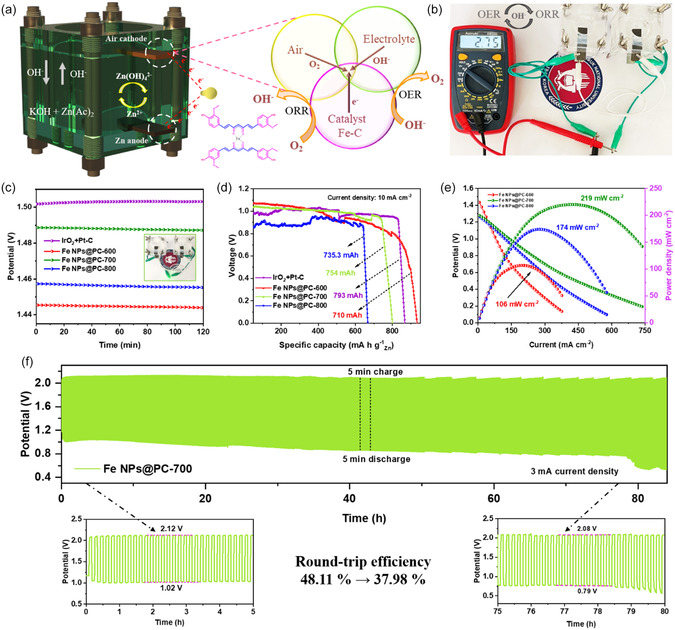
A zinc–air battery using a Fe NPs@PC air cathode in 6 M KOH with 0.2 M zinc acetate: (a) schematic of the cell setup, (b) an image of a volt meter connected with two Fe NPs@PC‐700 air‐cathode ZAB batteries with open‐circuit potential values, (c) open‐circuit potential curves (insert image; a green LED connected to two Fe NPs@PC‐700 air‐cathode ZABs), (d) specific capacity curves, (e) power density curves of Fe NPs@PC‐600, Fe NPs@PC‐700, Fe NPs@PC‐800 air‐cathode ZABs, (f) long‐term charge–discharge stability for an Fe NPs@PC‐700 air‐cathode ZAB (5 min of charging and 5 min of discharging).

The Fe NPs@PC‐600, Fe NPs@PC‐700, and Fe NPs@PC‐800 exhibited effective ORR/OER bifunctional catalytic properties. Construction of a ZAB confirmed its usefulness. **Figure** **4**a is a structural schematic of the ZAB. The primary components of a ZAB are the electrolyte, zinc‐plate anode, and air‐electrode cathode. Electrochemical oxidation of the zinc plate on the anode results in the production of electrons, which generate a current.^[^ ^67^ ^]^ On the cathode, catalytic ORR occurs at the air electrode in the center of the ZAB. First, oxygen from the surrounding air flows through the air electrode's breathable layer. It then diffuses to the catalytic layer, where it connects with the electrolyte and begins a three‐phase electrochemical reduction reaction.^[^ ^68^ ^]^

(3) $${\text{Cathode reaction: O}}_{2}{\text{+2H}}_{2}{\text{O + 4e}}^{-}⇌{\text{4OH}}^{-}\left(E^{0}\text{ = 0}\text{.4 V vs}\text{. SHE}\right)$$

(4)

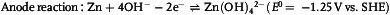

(5) $${\text{Overall reaction: Zn+½ O}}_{2}⇌\text{ZnO }\left(E^{0}\text{ = + 1}\text{.65 V}\right)$$

Equation ((3), (4))–(5) express how the produced hydroxyl ions move from the zinc anode to the air cathode to complete the cell reaction. The subsequent electrochemical reactions between the anode and cathode in solutions that are alkaline can be used to describe this entire discharge process.^[^ ^2^, ^3^ ^]^

A voltmeter was used to measure voltage in electrical circuits or electronic devices. Figure 4b shows the increase in oxygen in ZABs with an Fe NPs@PC‐700 electrocatalyst and a voltage of 2.75 V for two ZABs connected with a voltmeter. A light‐emitting diode with a rated voltage of ≈2.75 V was powered by dual liquid‐state ZABs linked in series (insert, Figure 4c). An open‐circuit voltage of 1.485 V was recorded and remained constant for 120 min (Figure 4c). Next, using galvanostatic studies at 10 mA cm^−2^ based on the mass reduction of zinc plates, the specific capacity and power density were determined. The specific capacity of the Fe NPs@PC‐700 based ZAB was 754 mA h g^−1^_Zn_, which is lower than that of the Pt/C + IrO_2_, Fe NPs@PC‐600, and Fe NPs@PC‐800‐based batteries in Figure 4d. This extremely porous bio‐carbon‐assisted Fe NPs catalyst outperformed a recently described catalyst in a ZAB due to its cost and environmental friendliness (Tables S1 and S2 and Figure S20c, Supporting Information). The increased OER/ORR behavior indicated that the peak power density of Fe NPs@PC‐600 (106 mW cm^−2^), Fe NPs@PC‐700 (219 mW cm^−2^), and Fe NPs@PC‐800 (174 mW cm^−2^) were significantly near than that of Pt/C + IrO_2_ (Figure 4e).

Battery cyclability was also tested using a high current density over an 80 h cycle that included 5 min of charging and 5 min of discharging at 3 mA current density. The Fe NPs@PC‐700‐based ZAB initial charge–discharge voltage differential and round‐trip efficiencies at a current density of 3 mA are shown in Figure 4f. By examining the galvanic charge–discharge curves, which involved 5 min of discharging and 5 min of charging each cycle, the battery cyclability was investigated by using Fe NPs@PC‐600 and Fe NPs@PC‐800 (see Figure S19a,b, Supporting Information). Fe NPs@PC‐700‐based Zn–air battery shows good cyclic stability with improved round‐trip efficiency from initial (48.1%) to final (37.9%). These results unequivocally demonstrated the feasibility of assembling a rechargeable ZAB with enhanced performance using Fe NPs@PC‐700 as an air‐electrode catalyst (Table S2, Supporting Information). For actual use of highly porous, pollutant‐free, and earth‐abundant non‐noble metals, it is crucial to examine the cycle stability at large capacity. For additional clarification, Figure S20c, Supporting Information, displays a higher power density in comparison to recently published articles.

Furthermore, structural deformation for the cathode catalyst of Fe NPs@PC‐700 electrode has been examined following ZABs charge–discharge cycle stability. The TEM results, demonstrating decreased structure degradation after ZABs stability due to persistent Fe NPs production with bio‐carbon support, are confirmed by steady crystal plane (110) and specific EDS elemental mapping (Figure S21, Supporting Information). According to recent studies, the formation of zinc dendrites and carbonates can be inhibited by aqueous electrolytes with almost neutral potential.^[^ ^8^, ^69^ ^]^ After discharging and following precipitation, high ZnO concentrations generate extra zinc hydroxide, increasing the resistance of the zinc electrode to passivation.^[^ ^3^, ^70^ ^]^ When zincate ions are reduced to zinc, oxygen is released (see Figure S22, Supporting Information). One of the most significant barriers to the growth of rechargeable ZABs is the time‐dependent characteristics of excessive saturation by zinc ions in an alkaline solution (see in Equation 6 and 7).^[^ ^3^ ^]^

(6)

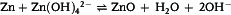

(7) $${\text{Zn+2OH}}^{-}⇌{\text{ZnO + H}}_{2}{\text{O + 2e}}^{-}\left(E_{n}\text{ = }-1\text{.25 V vs}\text{. SHE}\right)$$

## 5 Density Functional Theory of Fe NPs@PC Catalyst

**Figure 5:**
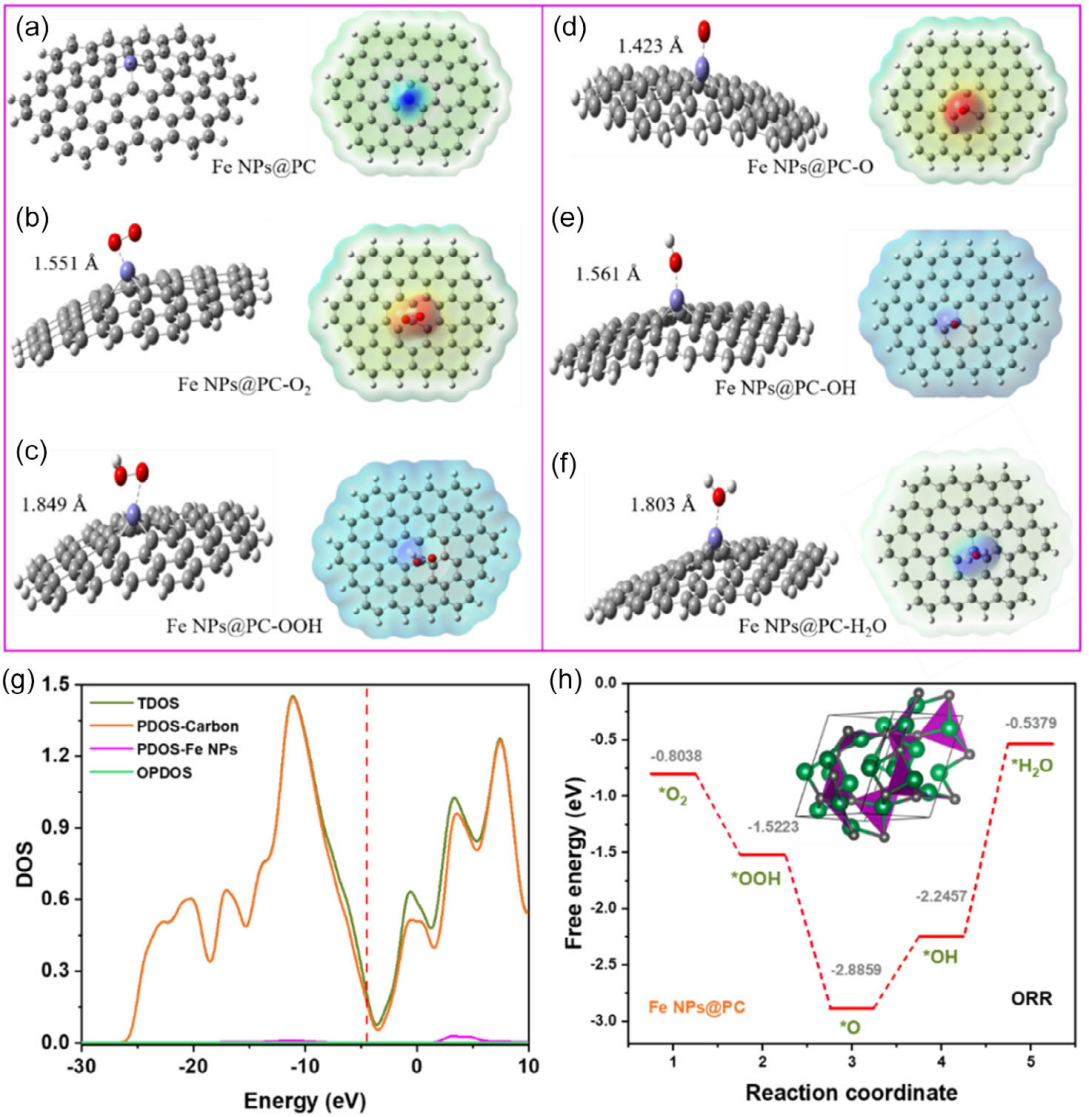
(a–f) On the Fe‐doped surface, the electrostatic charges of the *O_2_, *OOH, *O * OH, and *H_2_O molecule bond length were absorbed by the optimum structure. (g) The total density of state, partial density of state, and overlap density of states of carbon and Fe NPs. (h) Diagrams of the ORR Gibbs free energy of Fe NPs@PC and their adsorption intermediates (*O_2_, *OOH, *O * OH, and *H_2_O).

Exceptional electrochemical properties of porous bio‐carbon materials that contain Fe NPs@PC were studied further using DFT. To mimic the experimental studies, a graphene sheet model was prepared using 96 carbon atoms with terminal hydrogen atoms. All computational calculations were carried out in the Gaussian 16 software suite.^[^ ^71^ ^]^ A graphene sheet was optimized at a Hartree–Fock level of theory in the gas phase.^[^ ^72^, ^73^ ^]^ An optimized structure of the graphene sheet doped with Fe atoms was used to determine the suitable, stable saddled point with the lowest energy for an optimized structure. In the Fe‐doped optimized structure, the Fe atom disrupted the symmetrical structure of the graphene sheet. Adsorbents such as O, O_2_, OH, OOH, and H_2_O were placed on the Fe‐doped carbon sheet and optimized the structure (**Figure** **5**a–f). The respective electrostatic potential is also depicted in Figure 5a–f. The O_2_, OOH, O, OH, and H_2_O molecules were placed on the Fe‐doped surface, and the optimized structure shows respective bond lengths of 1.551, 1.849, 1.423, 1.561, and 1.803 Å.

The total density of state (TDOS), partial density of state (PDOS), and overlap density of states were used at the B3LYP/6‐31 g (d, p) level in the gas phase. The TDOS observed for the sum of density contributions at the atomic level shows each atomic orbital using the PDOS calculation. Multiwfn 3.4.1 was used to plot the TDOS graph. The fragments of graphene, Fe, and H_2_O were considered as fragments to plot the TDOS, with the results shown in Figure 5g. The *y‐*axis runs from −30 to 10 eV, whereas the vertical lines on the *x‐axis* represent the HOMO level. In the graph, pink, blue, red, and light blue represent the PDOS of fragments of graphene, iron, and water, respectively. Overall, the strength of the electronic interaction, PDOS, and the resulting changes in the TDOS generally increase in the order of H_2_O < OH < OOH < O < O_2_ (a detailed discussion is presented in Figure S23, Supporting Information).

According to Norskov and colleagues, computational hydrogen electrode theory indicates that every elementary ORR step entails a change in free energy (Δ*G*). Adsorption intermediates *O2, *OOH, *O * OH, and *H_2_O are used to optimize the catalyst's adsorption structures, which are shown in Figure S24, Supporting Information. Figure 5h displays a Gibbs free‐energy diagram, which shows clearly that Fe NPs@PC–O exhibited greater overpotential. In contrast to the adsorption of Fe active sites of *O_2_, *OOH, *O * OH, and *H_2_O, the ORR overpotential measured for every active site of the catalyst of Fe NPs@PC indicates an ideal overpotential of −2.8 V at each active‐site Fe NPs@PC–O. Compared with Fe NPs@PC–OH and Fe NPs@PC–OOH, the active site of Fe NPs@PC–O exhibited greater overpotential, indicating that the addition of Fe NPs improved ORR activity. We have modeled with 3Fe and 5Fe on a graphitic carbon framework to mimic the 3 and 5 nm Fe clusters observed in TEM. Various oxygen‐containing species such as O, O_2_, OH, OOH, and H_2_O were allowed to interact with the 3Fe and 5Fe active sites of the graphitic carbon framework, and density functional theory (DFT) calculations were performed at the HF level of theory in the gas phase using the Gaussian 16^[^ ^71^ ^]^ software package. The 3Fe and 5Fe systems DFT experiments are explained in detail in the Supporting Information file in Figures S25–S28, Supporting Information.

## 6 Conclusions

We developed and demonstrated an efficient route to the synthesis of Fe NP catalysts on porous carbon using a straightforward reflux approach and a bio‐carbon substrate. TEM and XRD analyses of the Fe NPs@PC catalyst revealed that Fe NPs were widely distributed on the porous bio‐carbon substrate. Iron and carbon interactions in the Fe NPs@PC catalyst were confirmed by XAS and DFT studies. Rapid electron transfer from an electrode into the electrolyte caused effective redox catalytic activity for the ORR and OER. These findings highlight the superior performance of ZABs enabled by the atomic active sites of Fe NPs in Fe NPs@PC, outperforming recently reported bio‐carbon‐based materials. Last, this work suggests that energy conversion and storage systems can be enhanced by designing diverse bio‐carbon supports integrated with metal nanoparticles possessing strong catalytic activity.

## 7 Experimental Section

7.1

7.1.1

##### Materials

Commercial turmeric yellow (Sigma‐Aldrich), iron (III) chloride hexahydrate (Sigma‐Aldrich, ≥99%), urea (Daejung, 98.0%), ammonium fluoride (Sigma‐Aldrich, ≥98.0%), methyl alcohol (Sigma‐Aldrich, 99.5%), iridium (IV) oxide (Sigma‐Aldrich, 99.9%), platinum on graphitized carbon (Sigma‐Aldrich, 20 wt% loading), potassium hydroxide (Sigma‐Aldrich, 95.0%), and deionized water were prepared in the laboratory.

##### Methods: Synthesis of Fe NPs@PC

Using a straightforward and cost‐effective reflux technique, metal NPs were produced on a highly porous bio‐carbon surface. In brief, 50 mg of turmeric yellow was dissolved in 50 mL of methanol and agitated for 1 h at room temperature. The mixture was stirred ultrasonically during dropwise addition of 4 mmol of iron (III) chloride hexahydrate and left to stand for 10 min. The sonicated mixture was then stirred for 2 h at room temperature and combined with 4 mmol of a urea solution and a small amount of ammonium fluoride as a structural tuning agent while being stirred constantly. Next, the solution was placed into a reflux system and stirred overnight at 130 °C. It was then centrifuged with a DI water/ethanol solution, and the obtained powder was pyrolyzed at 600 °C, 700 °C, and 800 °C for 2 h in a N_2_ atmosphere to produce the Fe NPs@PC‐600, Fe NPs@PC‐700, and Fe NPs@PC‐800 catalysts. The mechanism of synthesis involving the Fe NPs catalyst on carbon materials is described by Equation ((8), (9), (10))–(11) and summarized as Fe(redox reaction) = Fe^3+^ − Fe^2+^ − Fe_2_O_3_ − Fe_3_O_4_.^[^ ^74^, ^75^ ^]^ Example materials include hematite, magnetite, and iron in both ferric and ferrous states.

(8) $${\text{Fe}}_{\text{(s)}}↔{\text{ Fe}}^{\text{2+}}{\text{+ 2e}}^{-}\left(\text{oxidation}\right)$$

(9) $${\text{Fe}}^{\text{3+}}{\text{+ 3e}}^{-}↔{\text{Fe}}_{\text{(s)}}(\text{reduction})$$

(10) $${\text{2Fe}}_{\text{(s)}}{\text{+ 3H}}_{2}O↔{\text{ Fe}}_{2}O_{3}{\text{+ 6H}}^{+}{\text{+ 6e}}^{-}$$

(11) $${\text{Fe}}_{2}O_{\text{3(s)}}{\text{+ 3C}}_{\text{(s)}}\to {\text{ 2Fe}}_{\text{(s)}}{\text{+ 3CO}}_{\text{(g)}}$$

## Supporting Information

Supporting Information is available from the Wiley Online Library or from the author.

## Conflict of Interest

The authors declare no conflict of interest.

## Supporting information

Supplementary Material

## Data Availability

The data supporting the findings of this study are available within the article and its supplementary Information. Source data are provided in this paper.
