# Supplementary material for: Active‐Site Interactions in a Synergistic Porous Structured Fe Nanoparticle–Carbon Electrocatalyst for Enhanced Redox Reactions in Alkaline Zn–Air Batteries
Source: Small Sci. 2026 Feb 13;6(2):e202500448. doi: 10.1002/smsc.202500448 (PMC12903980; doi:10.1002/smsc.202500448)
Supplement: Supplementary file 1 — Supplementary Material [file SMSC-6-e202500448-s001.pdf]

# Supporting Information

## Active-Site Interactions in a Synergistic Porous Structured Fe Nanoparticle–Carbon Electrocatalyst for Enhanced Redox Reactions in Alkaline Zn-Air Batteries

Ramasamy Santhosh Kumar[a], Pandian Mannu[b], Venkatesan Srinivasadesikan[c], Narayanamoorthy Bhuvanendran[d], Chung-Li Dong[b], Dong Jin Yoo[a,e*]

[a]Graduate School, Department of Energy Storage/Conversion Engineering (BK21 FOUR), Jeonbuk National University, Jeonju, Jeollabuk-do, 54896, Republic of Korea

[b]Research Center for X-ray Science, Department of Physics, Tamkang University, Tamsui, 25137 Taiwan.

[c]Department of Chemistry, School of Science and Humanities, Vignan's Foundation for Science, Technology and Research, Vadlamudi, Guntur, Andhra Pradesh 522213, India.

[d]Department of Environmental Science and Engineering, SRM University AP, Amaravati 522240, Andhra Pradesh, India.

[e]Department of Life Science, R&D Education Center for Whole Life Cycle R&D of Fuel Cell Systems, Hydrogen and Fuel Cell Research Center, Jeonbuk National University, Jeollabuk-do, 54896, Republic of Korea

*Corresponding Email ID: djyoo@jbnu.ac.kr

**Materials characterization**

Morphological characterization of all manufactured electrocatalysts was conducted using a high-resolution transmission electron microscope and field emission scanning electron microscope with energy dispersive X-ray spectroscopy (EDS) (SUPRA 40 VP; Carl Zeiss, Germany) and HR-TEM (JEM-ARM200F, JEOL). X-ray diffraction (XRD) patterns of the produced electrocatalysts were evaluated using a PANalytical (X'PERT-PRO Powder) (model) and Cu K radiation (λ = 0.154 nm). Inductively coupled plasma-optical emission spectrometry (ICP-OES) was conducted using a Thermo Fisher Scientific iCAP 7000 series to assess the loading of Zn and Co. We used high-performance 3D mapping Imaging Raman spectroscopy with NANO PHOTON (RAMAN Touch) equipped with a 532 nm helium-neon laser to measure the Raman spectrum of all manufactured electrocatalysts. An X-ray photoelectron spectrometer (XPS; Axis-Nova, Kratos Inc.) was used to analyze the chemical condition of materials as-obtained.

**Electrochemical characterization**

**For ORR measurements:** ORR catalyst activities were evaluated using a rotating ring-disk electrode rotator (RRDE-3A, ALS Co., Japan) fitted to a Gamry Reference 600 Potentiostat/Galvanostat/ZRA. Graphite rod, Ag/AgCl, and RDE (5 mm: 0.19625 $cm^2$) were employed as the counter, reference, and working electrodes, respectively. The reference electrode was calibrated against RHE for each experiment. A homogeneous ink was produced by dispersing 4 mg of catalyst and 20 μL of 5% Nafion solution in 1 mL of isopropyl alcohol and DI water (1:1) solution followed by sonicating the mixture for 60 minutes.

Next, 15 μL of catalyst ink were drop-coated onto a rotating disk electrode (RDE; 5.0 mm in diameter). For comparison, we obtained a commercially available 20 wt% Pt/C (Johnson-Matthey, UK), and catalyst ink was made using a similar technique and was coated on the RDE

electrode. Cyclic voltammetry (CV: scan rate 50 mV $s^{-1}$) and linear-sweep voltammetry (LSV: 10 mV $s^{-1}$) were carried out with a $N_2$ or $O_2$ saturated 0.1 M KOH electrolyte. Additionally, RDE rotation rates between 400 and 2800 rpm with 0.2 and 0.8 V vs. Ag/AgCl were used to quantify LSV. Before each ORR test, the electrolyte was bubbled with oxygen for 30 minutes. $O_2$ flow was kept constant to preserve $O_2$ saturation throughout the measurements. The chronoamperometric method was used to conduct a 55-hour durability test at a constant voltage of 0.3 V vs. Ag/AgCl and 1600 rpm in an $O_2$ saturated 0.1M KOH environment.

**2.1. Calculation for number of electron transfer during ORR**

Koutecky–Levich (K–L) plots were used to determine the number of electrons transferred at vari ous potentials. ($J^{-1}$ vs $\omega^{-1/2}$)

$$\frac{1}{J} = \frac{1}{J_L} + \frac{1}{J_K} = \frac{1}{B\omega^{1/2}} + \frac{1}{J_K} \qquad (5)$$

$$B = 0.62\, nF\, C_0\, D_0^{\frac{2}{3}}\, \nu^{-1/6} \qquad (6)$$

Where, J, $J_L$ and $J_k$ are measured current density, diffusion-limiting current density and kinetic –l imiting current density, respectively.

**F**= Faraday constant (F = 96485 C $mol^{-1}$)

**ω**= Angular velocity for RDE (rad $s^{-1}$)

$\boldsymbol{D_O}$ Oxygen diffusion co-efficient $1.9 \times 10^{-5}$ $cm^2\, s^{-1}$

$\boldsymbol{C_O}$ =Saturated oxygen concentration of $1.2 \times 10^{-3}$ mol $L^{-1}$ in 0.1 M KOH

**n** = number of electron transfer during the ORR

**ν** kinetic viscosity of the electrolyte (0.01 $cm^2\, s^{-1}$)

All the measured potential vs Ag/AgCl ($E_{Ag/AgCl}$) were convert in to potential vs standard RHE ($E_{RHE}$) by using following universal Nernst equation (7).

$$E_{RHE} = E_{Ag/AgCl} + 0.059pH + E^{\circ}_{Ag/AgCl} \qquad (7)$$

Where $E°_{Ag/AgCl}$ = 0.1976 at 25 C and pH of 0.1M (ORR measurement) and 0.1M KOH (OER measurement)

**For OER measurements:** A homogeneous ink was created by combining 3 mg of electrocatalyst with 5% Nafion in 30 L of a 1:1 isopropyl alcohol/DI water solution and sonicating the mixture for 60 minutes. An area of around 1 $cm^2$ of carbon paper was coated with the produced ink, which was then dried at 60°C for 12 h in a vacuum oven. Approximately 3 mg $cm^{-2}$ of active compounds were loaded. A catalyst ink of commercial $IrO_2$ (99.9%; Sigma-Aldrich) and Pt/C were made using a similar process and were coated on carbon paper for comparison tests. The OER electrochemical performance was examined using a novel three-electrode cell design that included a working electrode made of catalyst-coated carbon paper, a reference electrode made of Ag/AgCl, and a graphite road (counter electrode).

A fixed scanning rate of 1 mV $s^{-1}$ was used to record the OER polarization curves. The produced electrocatalysts were subjected to electrochemical impedance spectroscopy (EIS) measurements with a frequency range of 0.01 to $10^6$ HZ and a potential amplitude of 5 mV. The double-layer capacitance ($C_{dl}$) value of the produced catalyst was assessed using a scanning rate of 10 to 50 mV $s^{-1}$ in the non-Faradic section of the CV curve. The difference between the anodic and cathodic current densities represented the midpoint of the potential. Current densities had a slope that was twice as steep as $C_{dl}$ in relation to scan rate.

<u>Calculation of ECSA</u>

$$ECSA = \frac{C_{dl}}{C_s} \quad (8)$$

ECSA = Electrochemical active surface area (ECSA)

$C_{dl}$ = Double layer capacitance

$C_s$ = Specific capacitance (0.040 mF $cm^2$)

**Zinc-Air Battery Test**

Carbon paper foam was coated (≈1 cm2) with Cur-Fe optimized catalyst ink (Toray Carbon Paper, TGP-H-60, 19 × 19 cm, Alfa Aesar). A zinc-air battery was constructed using an air cathode, zinc foil (0.25 mm thick, Alfa Aesar, UK), and an electrolyte of 6 M KOH and 0.2 M Zn (CH3CO2)2. The catalyst loading was kept at 1 mg cm−2. Durability tests for long-term charge-discharge cycles were examined using a Gamry 600 electrochemical workstation. For comparison, a similar process was utilized to create an air cathode electrode made of Pt/C (20 wt.%) and IrO2 (99.9%; Sigma-Aldrich) using a weight ratio of 1:1 for commercial Pt/C and IrO2. Equations (1) and (2) were utilized to calculate the power density (mW cm−2) and specific capacity (mAh g−1) of zinc-air batteries using Pt/C + IrO2 and Fe NPs@PC as the air cathode.[1, 2]

Power density (mW $cm^{-2}$) = Voltage × current density (9)

Specific capacity (mAh $g^{-1}$) = current × service hours/weight of consumed Zn (10)

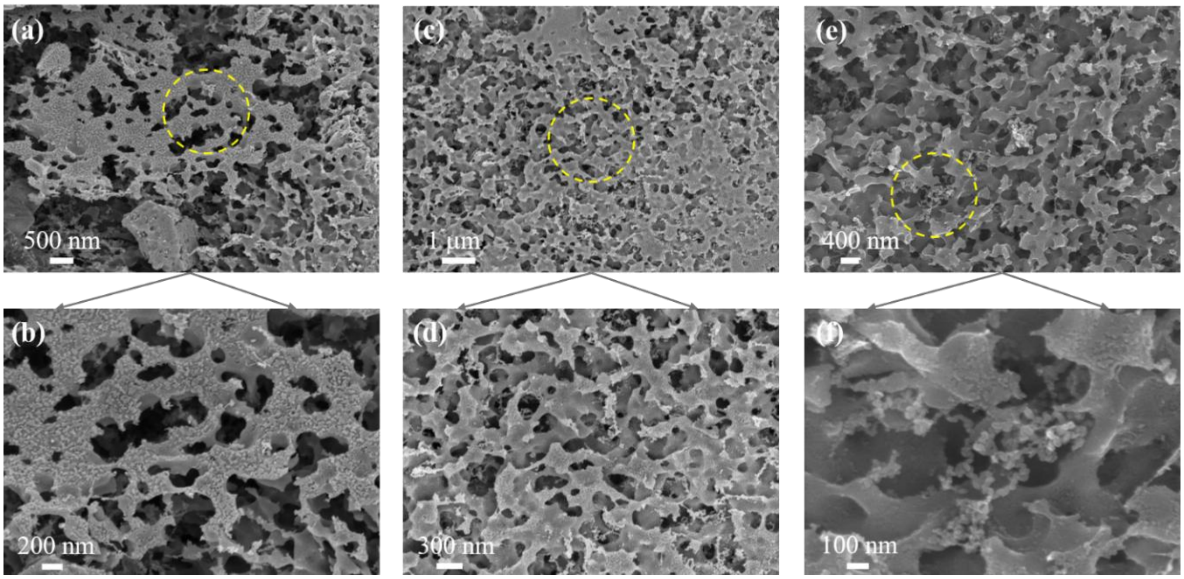


**Figure S1.** (a-f) SEM analysis of Fe NPs@C catalyst.

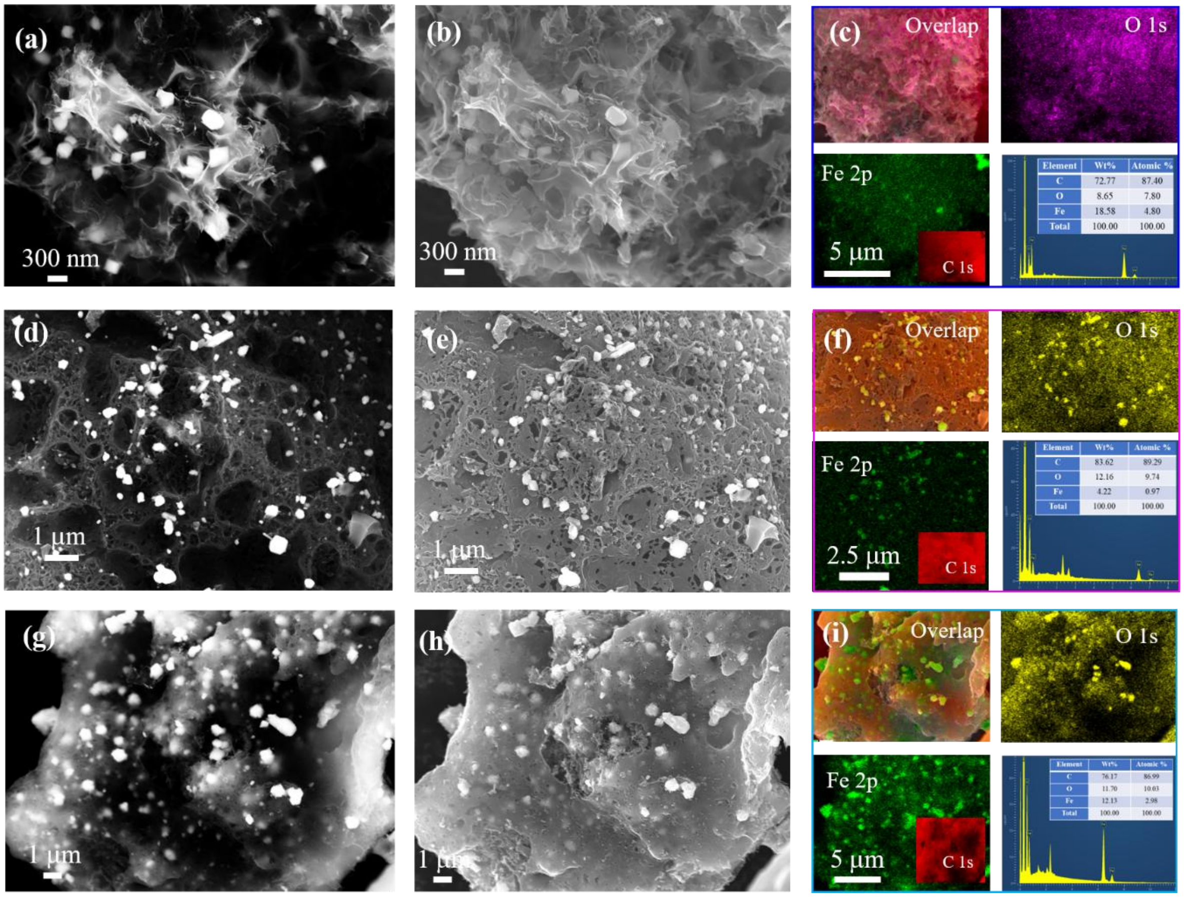


**Figure S2.** SEM image, HAADF-SEM, and EDS elemental mapping, and energy-dispersive X-ray spectroscopy (EDX) spectrum of (a, b, c) Fe NPs@PC-600, (d, e, f) Fe NPs@PC-700, and (g, h, i) Fe NPs@PC-800 composite (insert: elemental percentage and individual EDX-elemental images).

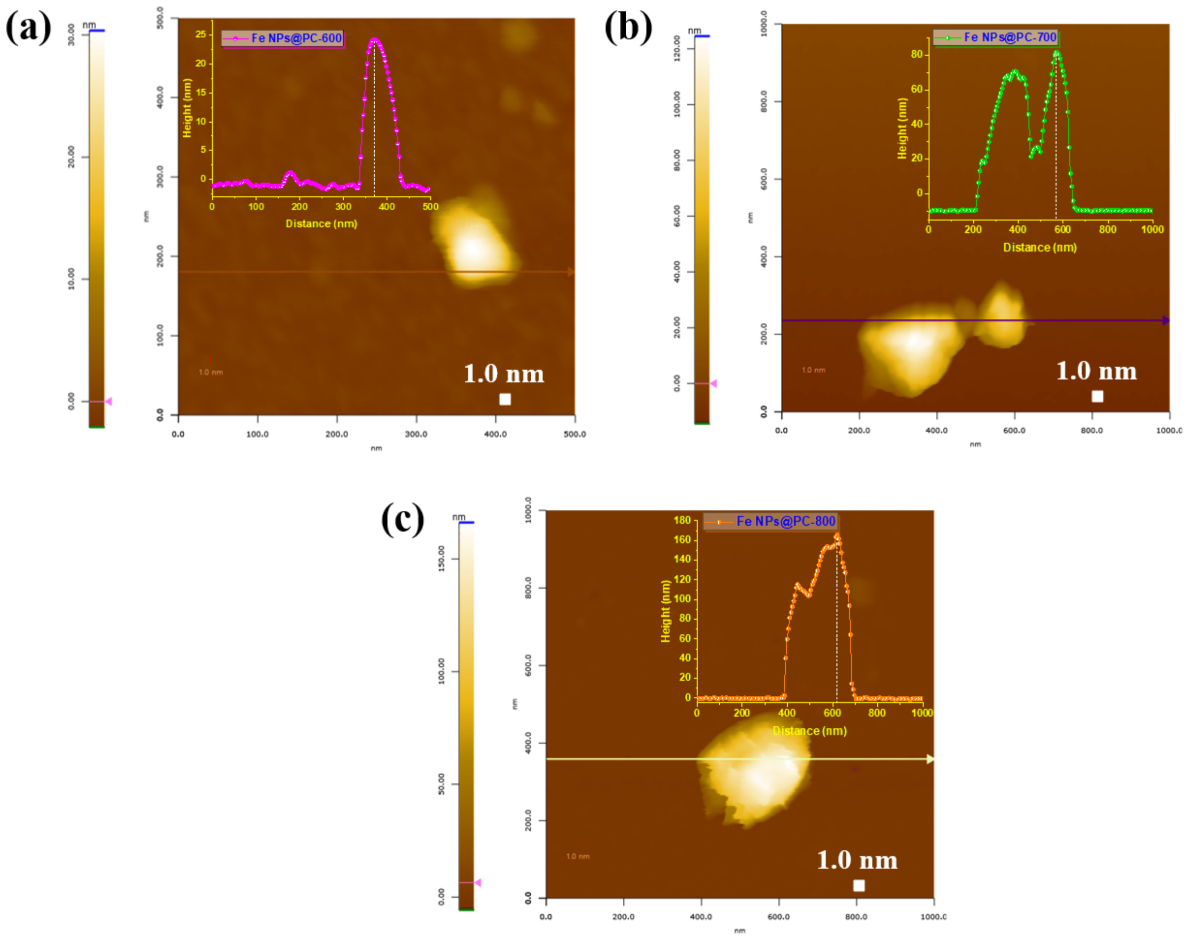


**Figure S3.** AFM analysis of (a) Fe NPs@PC-600, (b) Fe NPs@PC-700, and (c) Fe NPs@PC-800 composites.

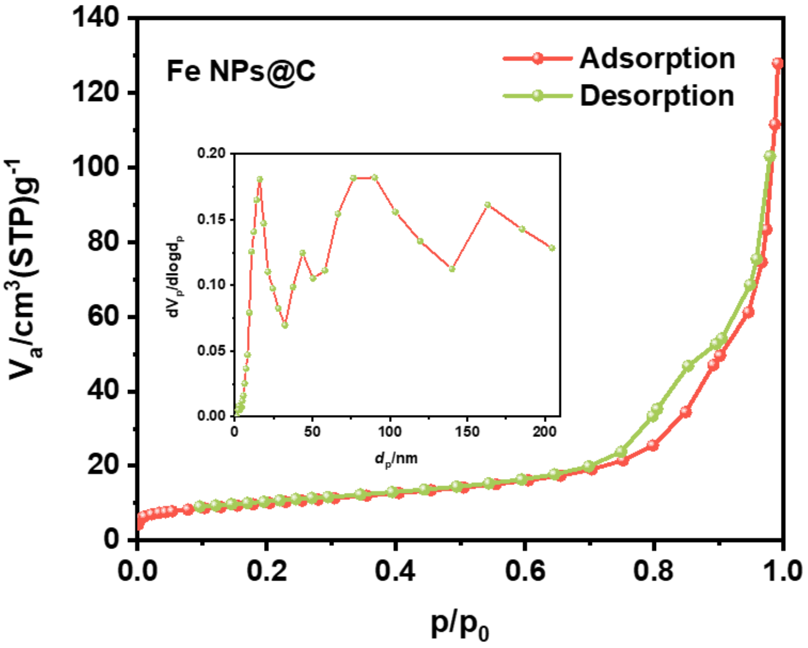


**Figure S4.** BET and insert image pore size distribution of Fe NPs@C catalyst.

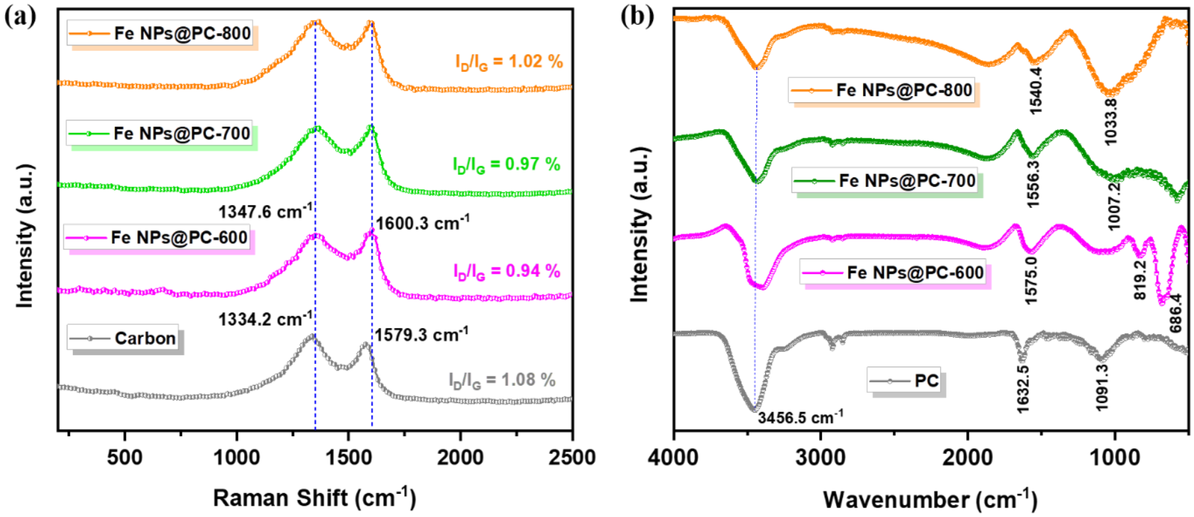


**Figure S5.** (a) Raman and (b) FT-IR spectra of biocarbon with Fe nanoparticles of Fe NPs@PC-600, Fe NPs@PC-700, and Fe NPs@PC-800 composites.

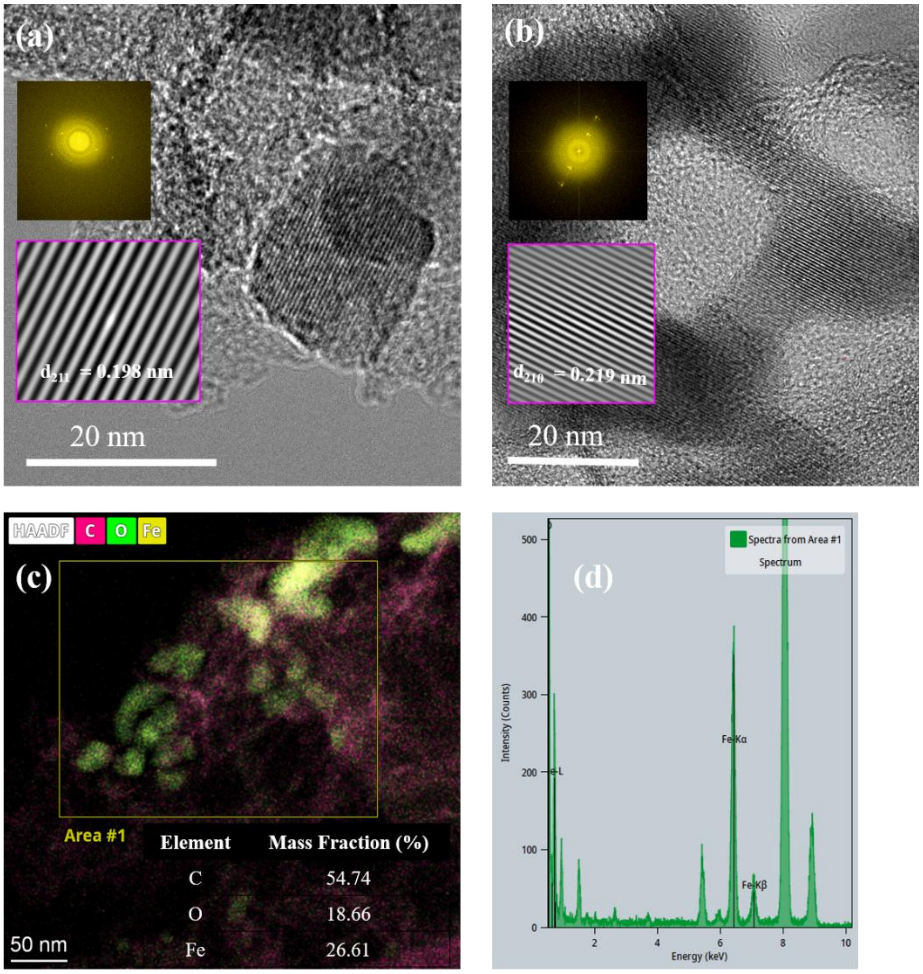


**Figure S6.** (a, b) HR-TEM images and (c, d) TEM-EDX mapping and EDX elemental spectrum of Fe NPs@PC-600 catalyst.

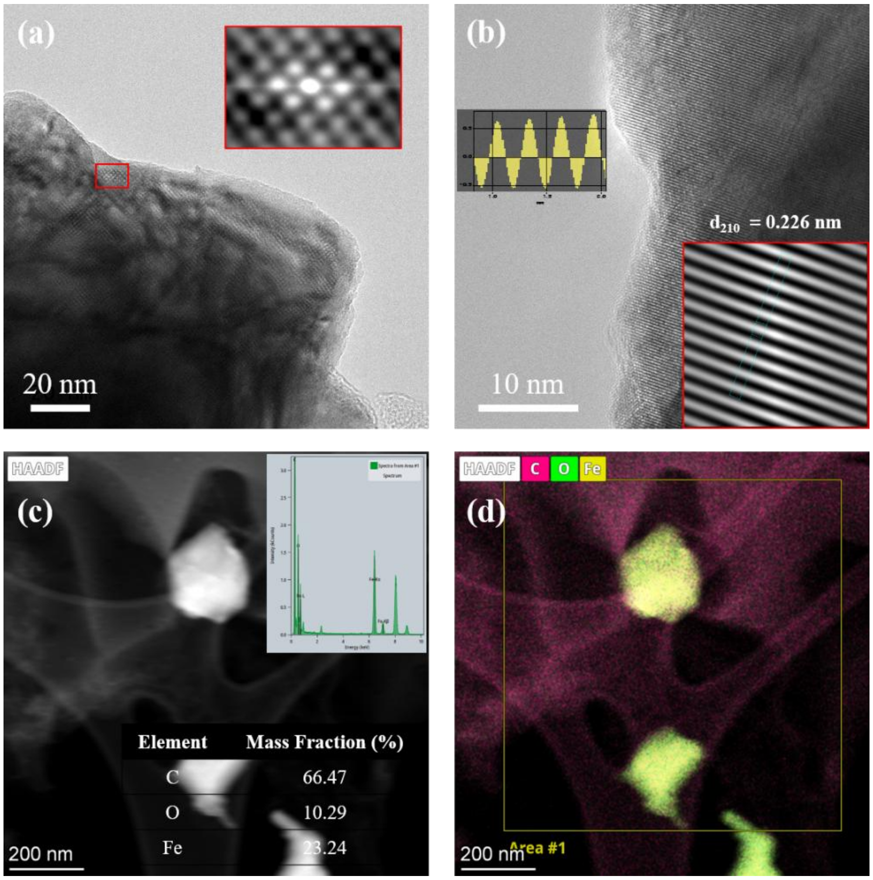


**Figure S7.** (a, b) HR-TEM images and (c, d) TEM-EDX mapping and EDX elemental spectrum of Fe NPs@PC-800 catalyst.

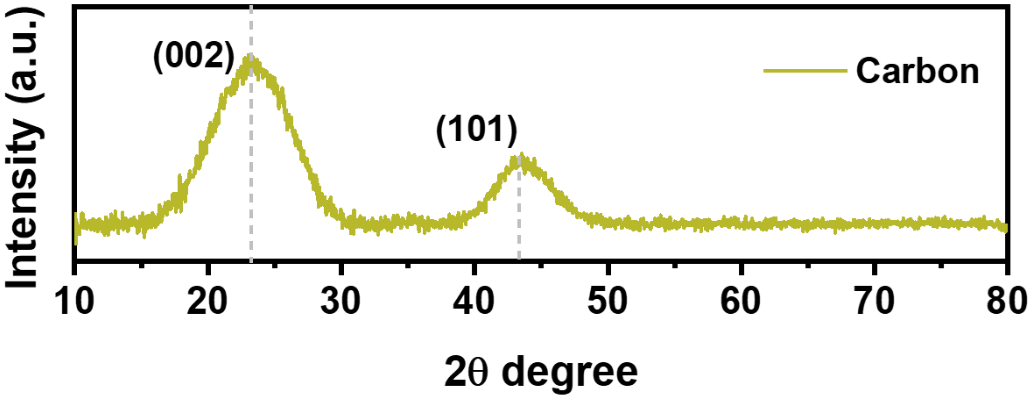


**Figure S8.** XRD analysis of Biomass-derived carbon.

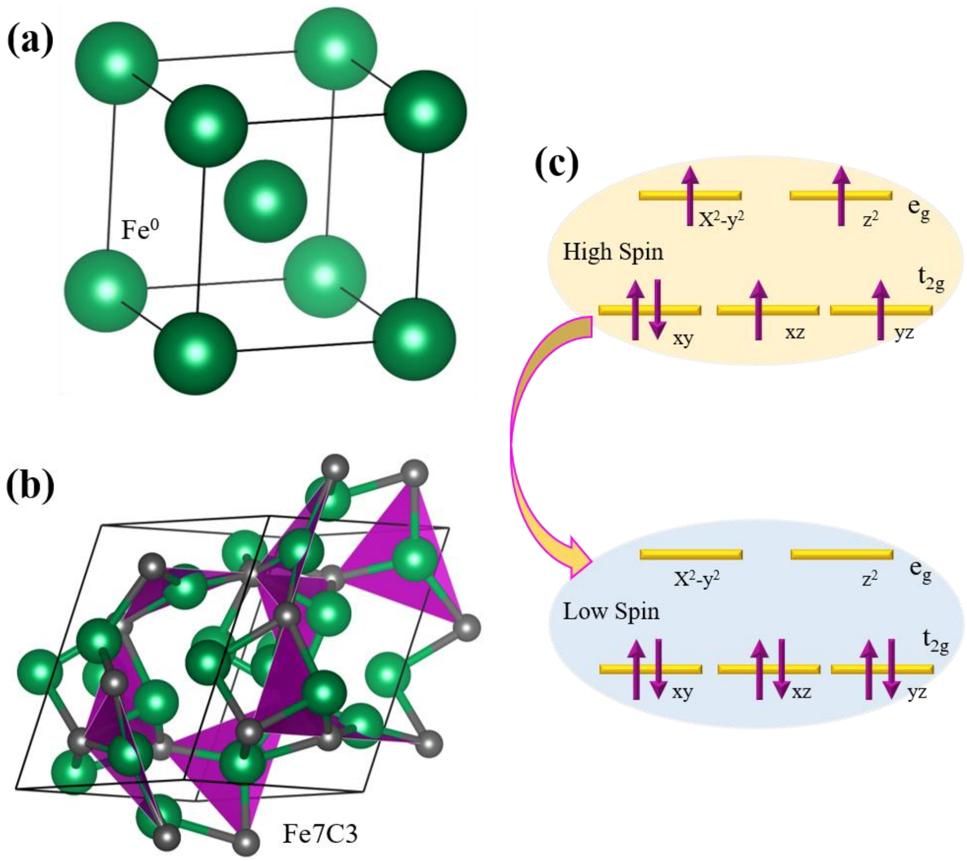


**Figure S9.** (a) Fe and (b) Fe7C3 crystal systems for following XRD analysis. (c) Fe(II) octahedral centers with ligand-field splitting and filling of the five d-orbitals, where a transition between a low-spin state (S = 0) and a high-spin state (S = 2) defines the spin crossover.

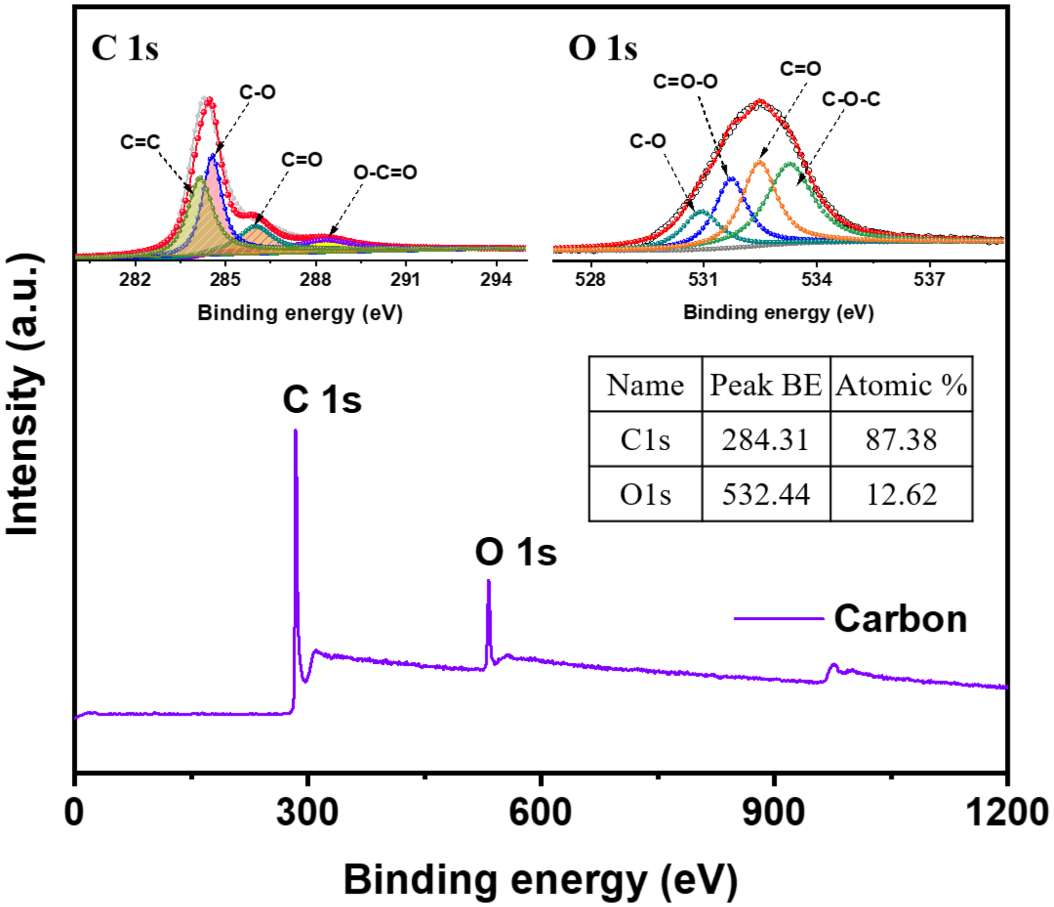


**Figure S10.** XPS survey spectrum of biomass-derived carbon composites.

**(a)**

| Name 600 | Peak BE | Atomic % | Name 700 | Peak BE | Atomic % | Name 800 | Peak BE | Atomic % |
|---|---|---|---|---|---|---|---|---|
| C1s | 284.09 | 80.97 | C1s | 284.53 | 90.47 | C1s | 284.52 | 87.56 |
| O1s | 530.61 | 14.72 | O1s | 531.87 | 8.23 | O1s | 530.22 | 9.05 |
| Fe2p | 710.49 | 4.31 | Fe2p | 710.84 | 1.3 | Fe2p | 710.88 | 3.39 |

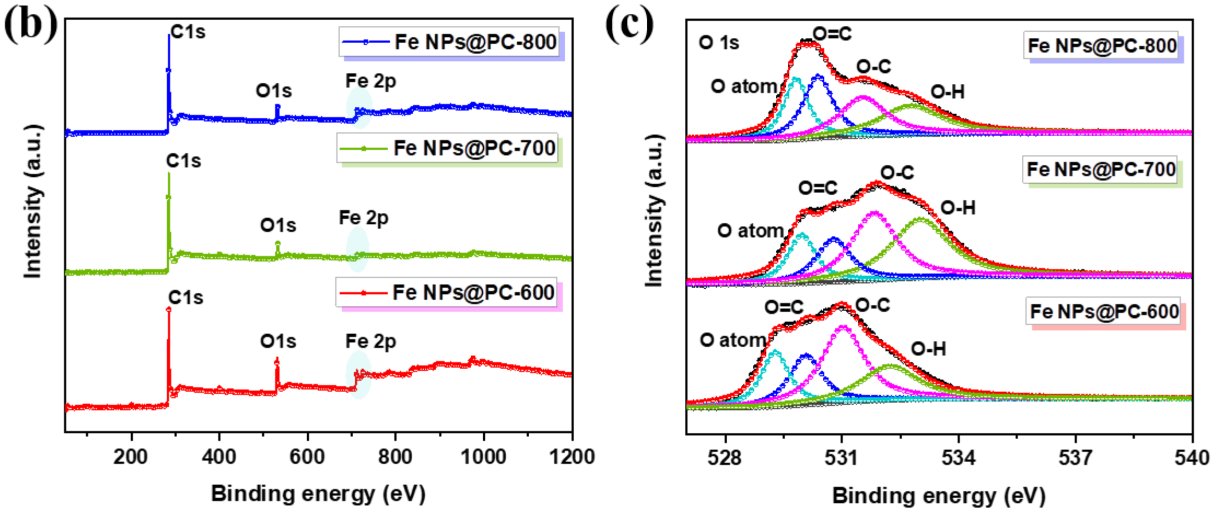


**Figure S11.** (a) XPS peak tables, (b) XPS survey spectrum, and (c) high-resolution O 1s XPS spectrum of Fe NPs@PC-600, Fe NPs@PC-700, and Fe NPs@PC-800 composites.

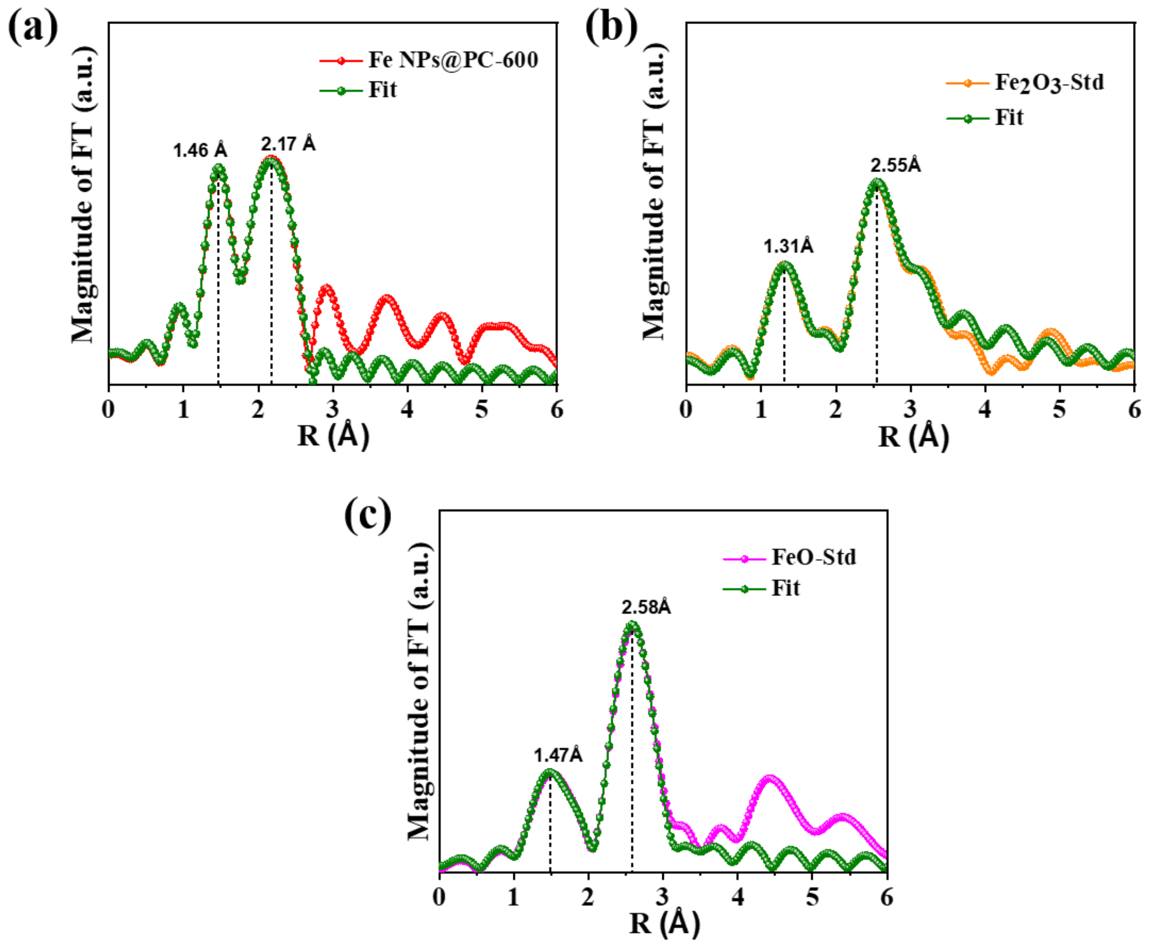


**Figure S12.** EXAFS peak fitting for Fe-C, FeO, and $Fe_2O_3$ catalysts.

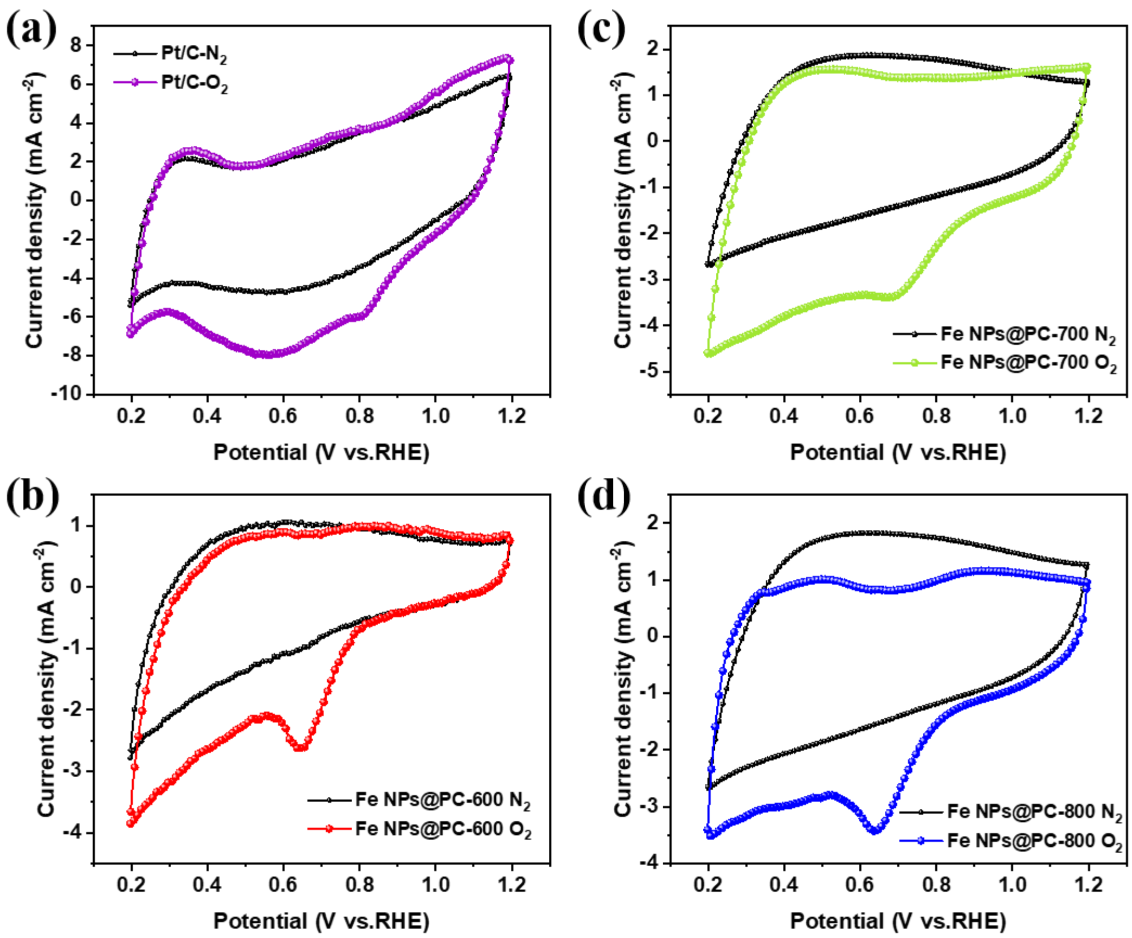


**Figure S13.** An ORR study in a 0.1 M KOH electrolyte solution: (a-d) CV curves at a 50 mV scan rate for Fe NPs@PC-600, Fe NPs@PC-700, and Fe NPs@PC-800 electrocatalysts.

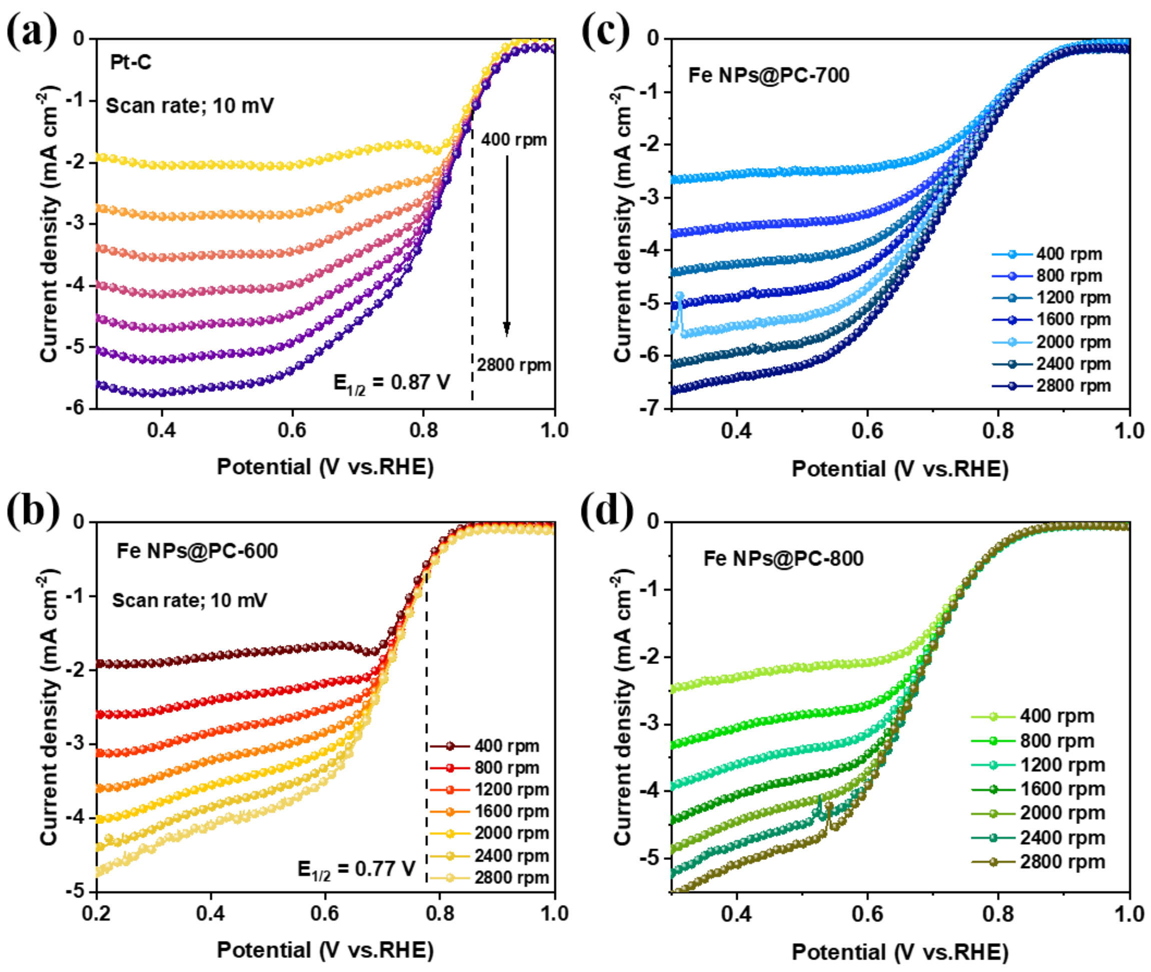


**Figure S14.** (a-d) LSV curves at rotation speeds of 400-2800 rpm for commercial Pt-C, Fe NPs@PC-600, Fe NPs@PC-700, and Fe NPs@PC-800 electrocatalysts, respectively.

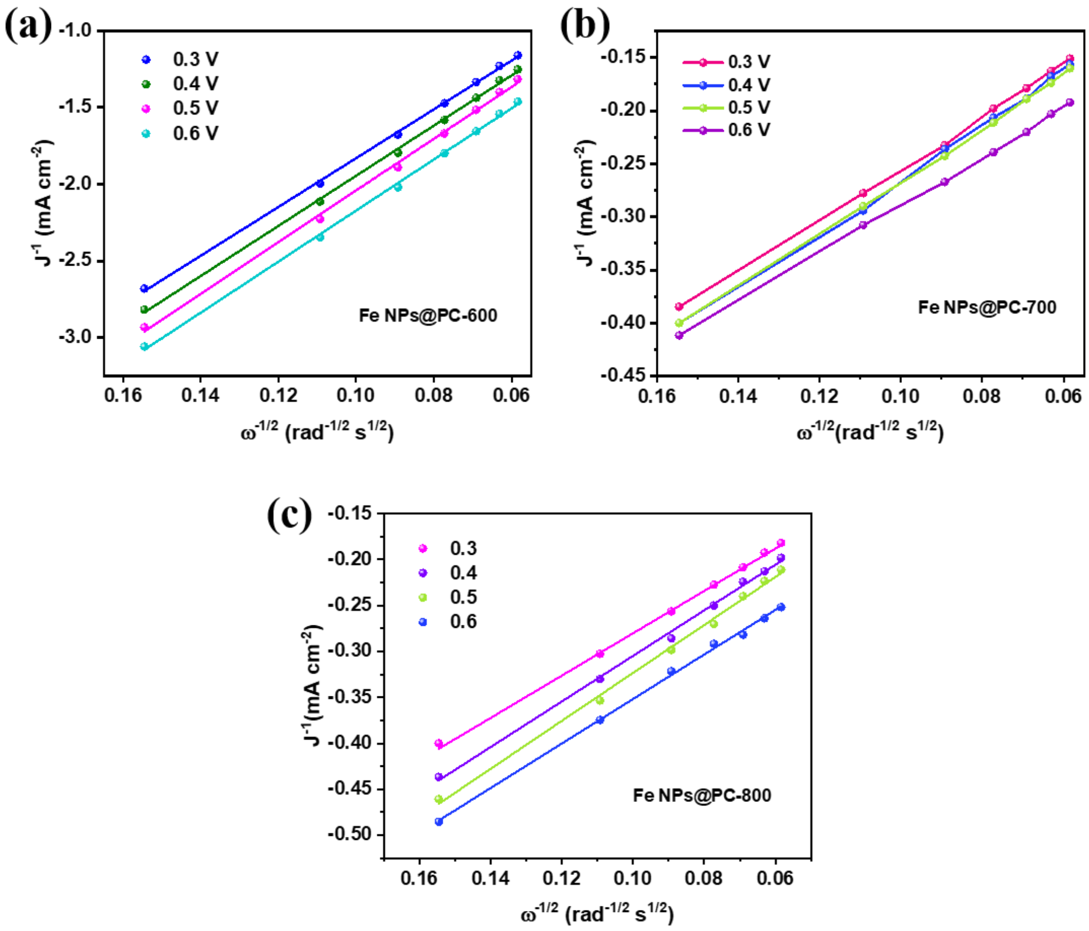


**Figure S15.** The K-L plots of Fe NPs@PC-600, Fe NPs@PC-700, and Fe NPs@PC-800 electrocatalysts, respectively.

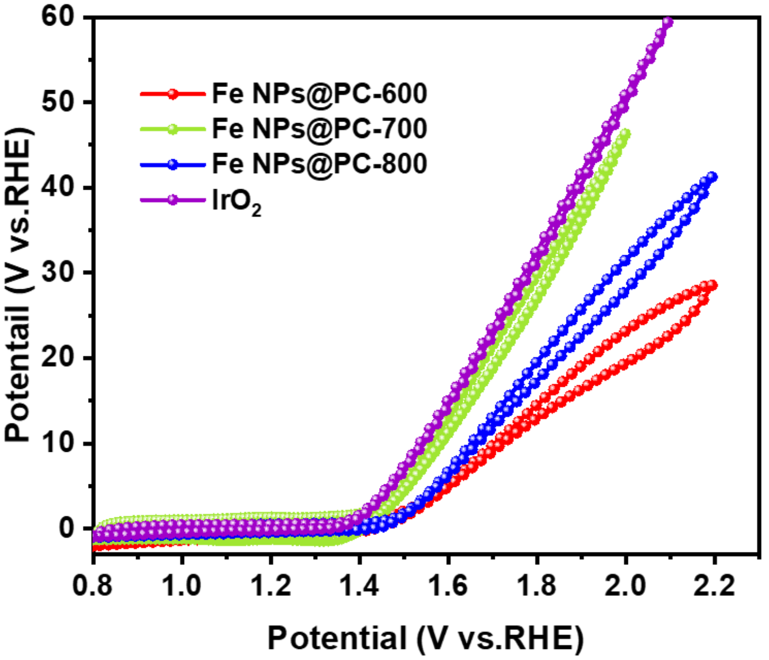


**Figure S16.** Cyclic voltammetry curves of $IrO_2$ and Fe NPs@PC-600, Fe NPs@PC-700, Fe NPs@PC-800 electrocatalyst at 25 mV scan rate in a 0.1 M KOH solution.

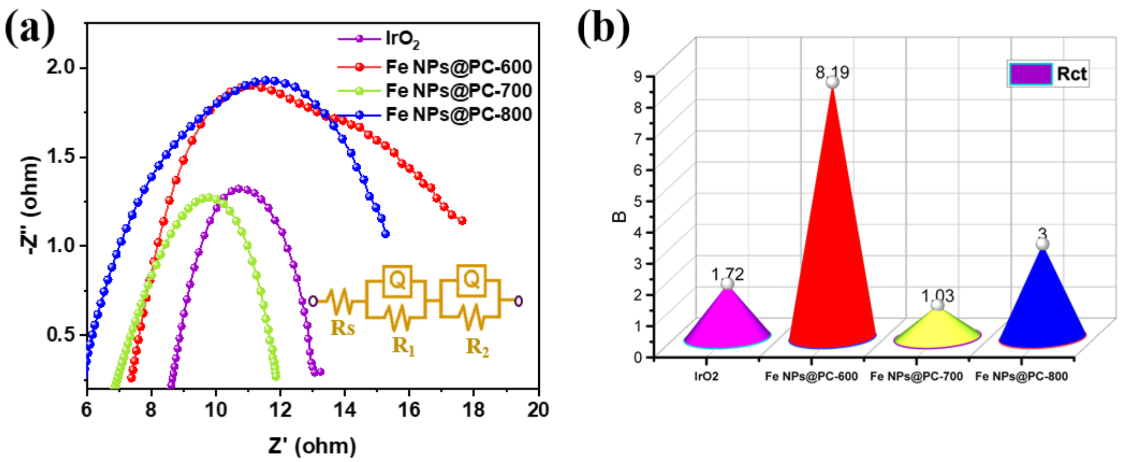


**Figure S17.** (a, b) Electrochemical impedance spectroscopy for measured and calculated curve for $IrO_2$ and Fe NPs@C-600, Fe NPs@PC-700, and Fe NPs@PC-800 electrocatalysts.

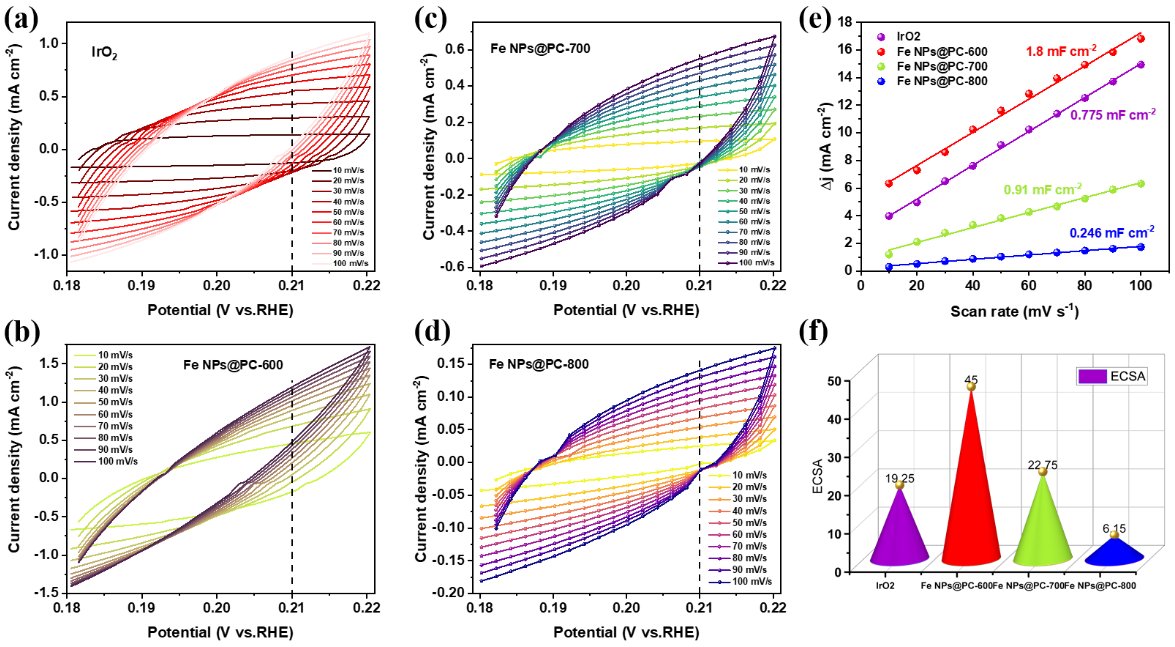


**Figure S18.** (a, b) Double layer capacitance ($C_{dl}$) for following different scan rates (10-100 mV/s) of $IrO_2$ and Fe NPs@C electrocatalysts, respectively. (f) bar chart diagram of active surface area and Rct values of $IrO_2$ and Fe NPs@C electrocatalyst.

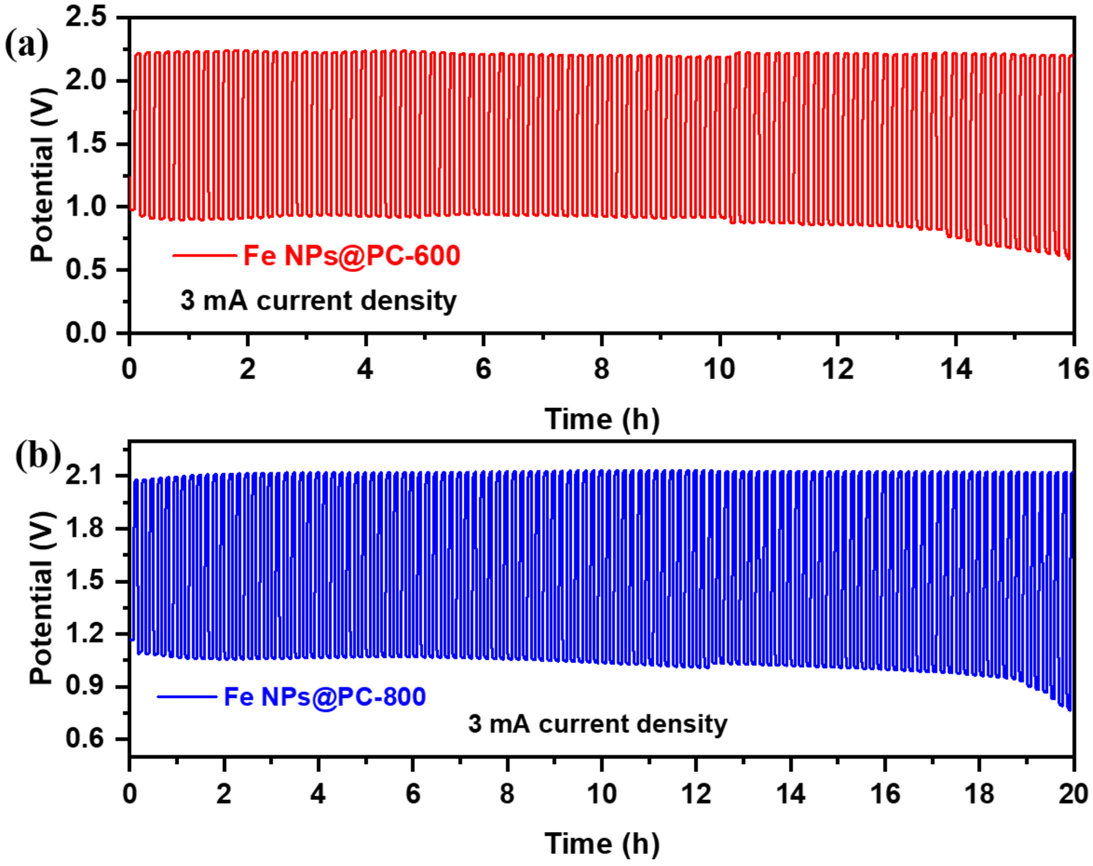


**Figure S19.** (a, b) long-term charge-discharge stability test at 5 mA current density of 10 minutes charging 10 minutes discharging for using Fe NPs@C air cathodes Zn-Air battery.

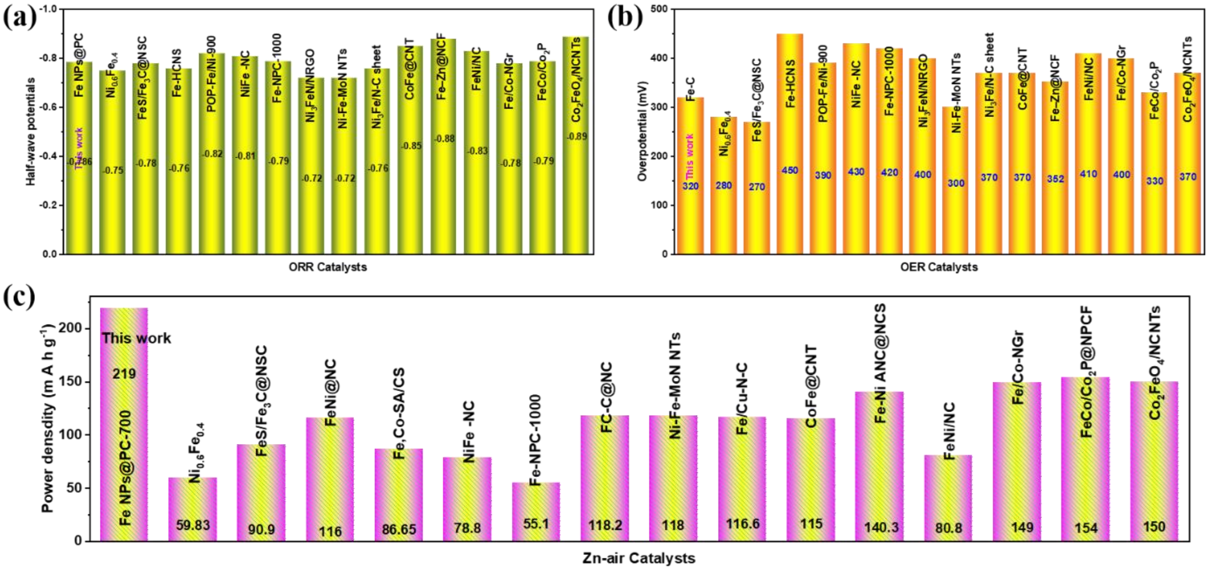


**Figure S20.** Comparison recent research articles of (a) half-wave potential of ORR study, (b) overpotentials of OER, and (c) power density of Zn-air battery performances.

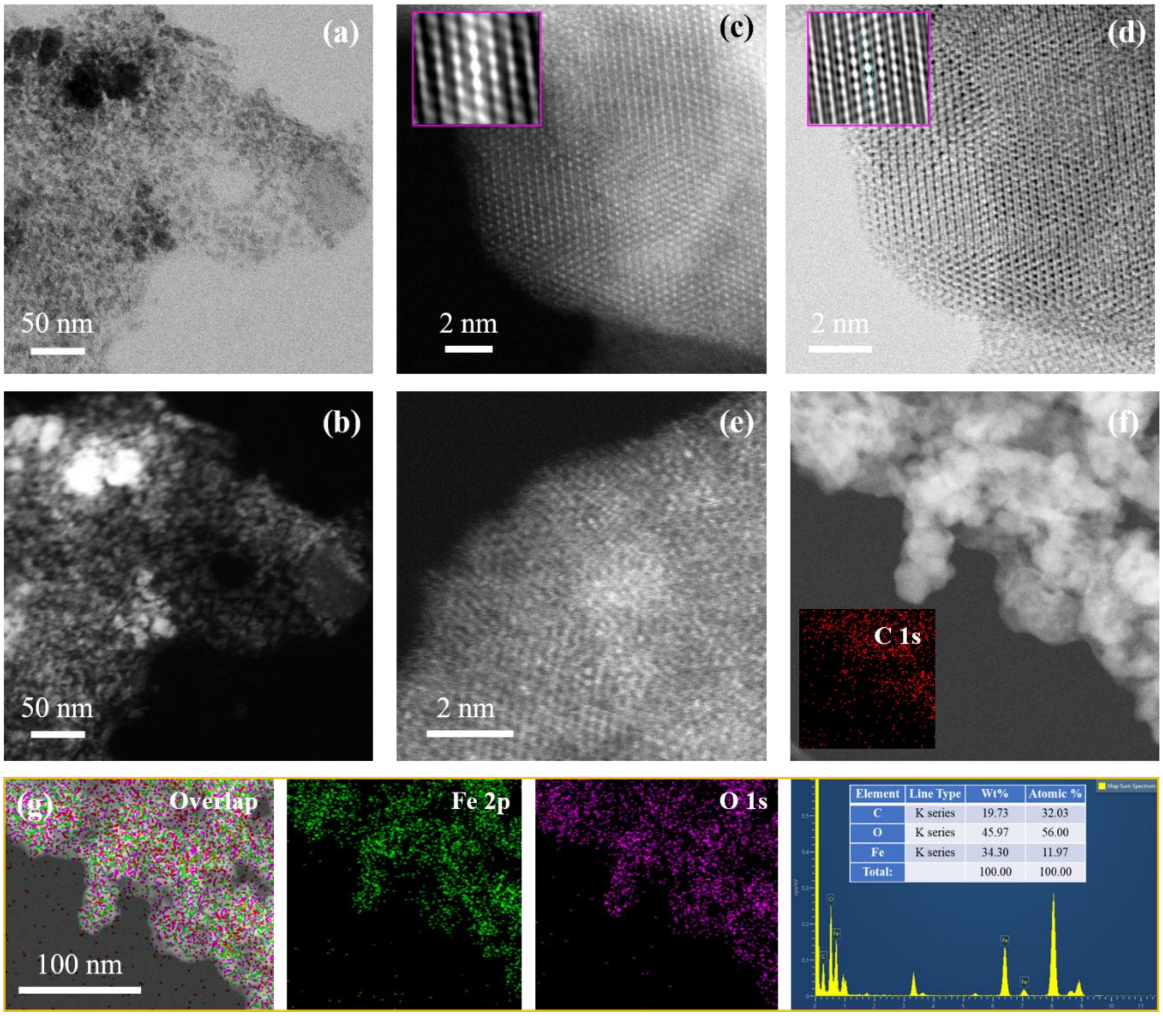


**Figure S21.** STEM, HR-TEM, and STEM-EDS elemental mapping for after Zn-air battery stability of Fe NPs@PC-700 air-cathode electrode.

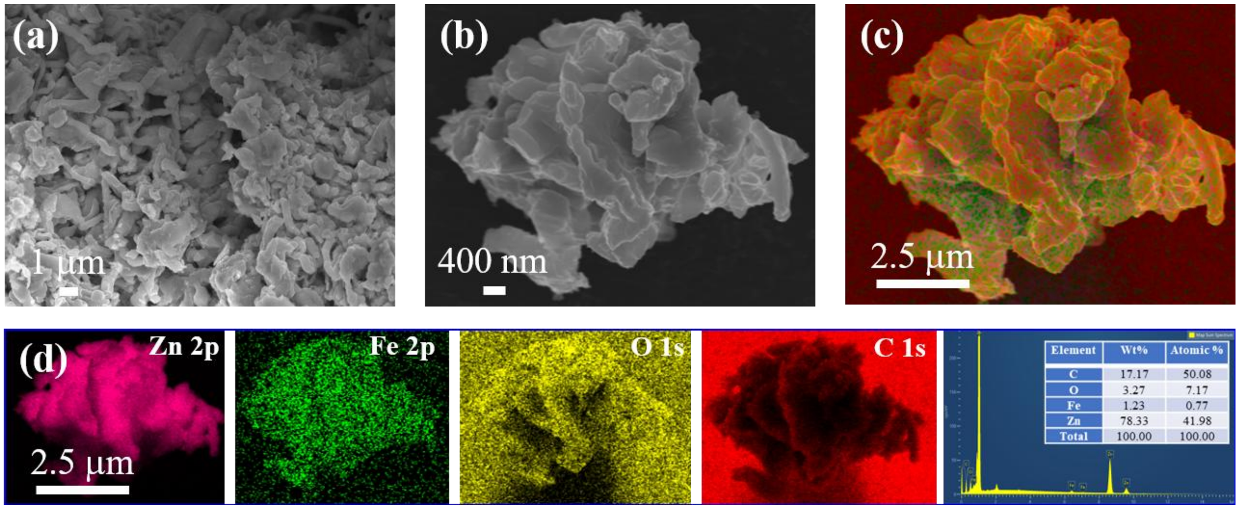


**Figure S22.** SEM and SEM-EDX elemental mapping for after Zn-air battery charge-discharge stability by using Zn plate air-anode electrode.

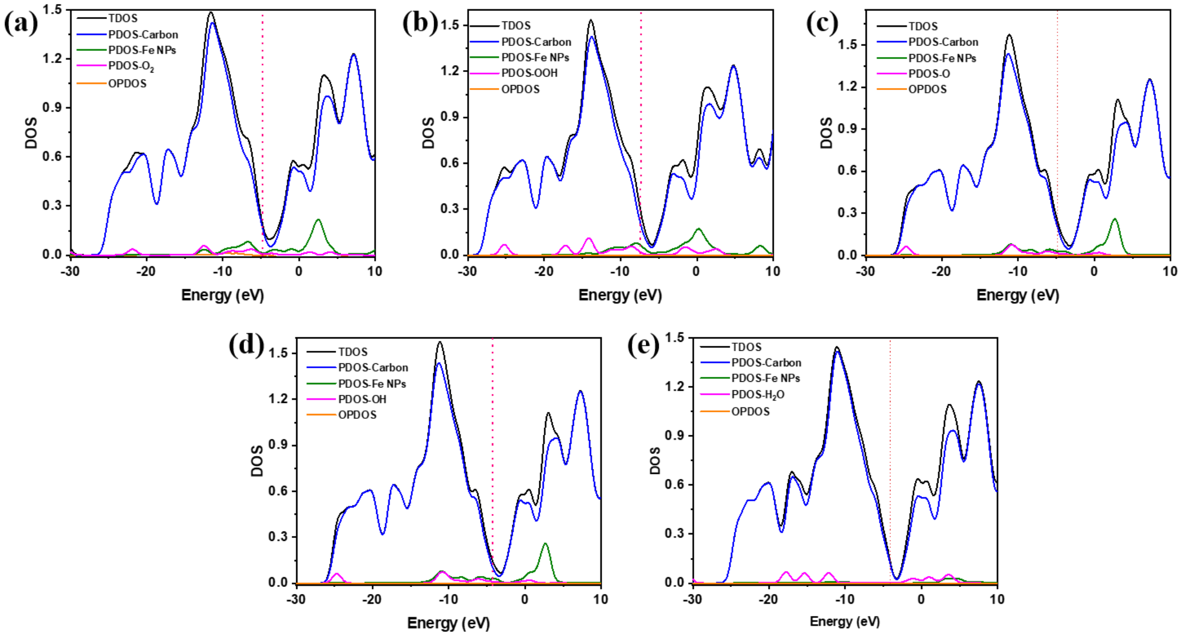


**Figure S23.** (a-e) The total density of state, partial density of state, and overlap density of states of carbon and Fe NPs and their adsorption intermediates (* $O_2$, *OOH, *O *OH, and* $H_2O$).

This figure presents the calculated Density of States for a system comprising graphene, iron, and

water. The plot displays the total density of states (TDOS) in black, the partial density of states (PDOS) projected onto graphene (red), iron(blue), and water (pink), as well as an orbital projected density of states (OPDOS) in green. The energy is plotted in electron volts (eV) relative to the Fermi level, which is indicated by the vertical dashed line at approximately-5eV. The left y-axis represents the density of states, while the right y-axis corresponds to the OPDOS.

The TDOS reveals a complex electronic structure across the plotted energy range (-30 eV to 10 eV). Several prominent peaks and valleys indicate the presence of numerous electronic states at specific energy levels. The TDOS shows significant density of states in the valence band region (negative energies relative to the fermi level) and extends into the conduction band region (positive energies). The fermi level appears to fall within a region of relatively low density of states, suggesting potential semiconducting or semi-metallic behavior for the overall system.

The PDOS for graphene (red line) exhibits a prominent and sharp peak centered around -10 eV reaching an approximate intensity of 1.40 states/eV or slightly higher, dominating the electronic structure at this energy level and in the conduction band around 6 eV, with an intensity of about 0.75 states/eV. The PDOS of iron (blue line) shows much weaker contributions, with a peak around 5 eV at approximately 0.05 states/eV indicating a less significant and more localized role for iron's electronic states. Finally, the PDOS of water (Pink line) remains low, confirming its minimal direct contribution to the electronic density of states in this system. These values highlight the dominant role of graphene in shaping the electronic properties, with iron contributing specific states at higher energies and water having a negligible direct impact on the electronic density of states as depicted.

The TDOS (black line) shows significant states in both the valence and conduction bands, with the fermi level around -5eV. The PDOS of graphene (red line) closely matches the TDOS,

highlighting graphene as the main contributor, especially around -10 eV and -2 eV. Iron's PDOS (blue line) display smaller, localized peaks at higher energies, indicating weaker hybridization with the graphene work.

The PDOS of the adsorbed hydroxyl (OH) group (pink line) shows a small but noticeable contribution, particularly in the valence band region around -10 eV -19 eV, indicating electronic interaction with the Fe-C substrate. The OPDOS (green line) displays low intensity, suggesting minor contributions from specific orbitals. Though small, the OH derived states reveal that adsorption alters the Fe-C electronic structure, potentially affecting its chemical and catalytic behavior.

This plot shows the electronic structure of an iron-decorated graphene (Fe-C) system with an adsorbed OOH group. The TDOS (black line) spans both valence and conduction bands, with the fermi level around -5 eV. The graphene PDOS (red line) dominates, with strong peaks near -13eV, -2eV and 4eV, while iron's PDOS (blue line) shows small, localized peaks at higher energies, indicating limited contribution and weak hybridization with the graphene.

The PDOS of the adsorbed OOH group (pink line) shows stronger contributions than simpler adsorbates, with distinct peaks around -6eV, -14eV, -17eV and -25eV indicating significant interaction with the Fe-C substrate. The OPDOS (green line) remains low, suggesting minimal orbital contribution. The presence of OOH- derived states highlights notable electronic structure changes in Fe-C, potentially enhancing its catalytic activity and reactivity towards OOH group.

This plot shows the electronic structures of an iron-decorated (Fe-C) system with an adsorbed Oxygen atom. The TDOS (black line), with the Fermi level near -5 eV, closely follows the graphene PDOS (red line), marked by peaks around -12eV and 8 eV, confirming graphene as the main contributor. Deviations from graphene indicate the impact of oxygen adsorption and iron

decoration on the electronic structure.

The PDOS of the adsorbed oxygen atom (pink line) shows distinct peaks around -6 eV, -11 eV, and -25 eV, indicating strong interaction and hybridization with the Fe-C substrate. Iron PDOS (blue line) contributes mainly in the conduction band (~ 4 eV and 3 eV), suggesting its role in mediating oxygen-graphene interactions. The OPDOS (green line) remains low, indicating minor orbital contributions. The findings highlight the significant impact of oxygen adsorption on Fe-C's electronic structure, relevant to its catalytic and reactive behavior towards oxygen.

The plot of the Fe-decorated graphene (Fe-C) with adsorbed $O_2$ shows the TDOS (black line) and PDOS of graphene (red line), with the Fermi level around -5 eV. Graphene's PDOS dominates, with peak around -10 eV, -2 eV (valance band) ad 6 eV (conduction band). Deviations in TDOS from graphene indicate the influence of Fe decoration and O2 adsorption on the electrostatic structure.

The PDOS of adsorbed O2 (pink line) shows peak at -4 eV, -12 eV and -21 eV (valence band), and around 3 eV and 5 eV (conduction band), indicating strong electronic interaction and hybridization with Fe-C. The Iron PDOS (blue line) contributes notably around 3 to 5 eV suggesting iron mediates the $O_2$-graphene interaction. The OPDOS (green line) shows low intensity, implying minimal contribution from the projected orbitals. Hence the $O_2$ adsorption significantly alters the electronic structure, highlighting its impact on the Fe-C system's catalytic properties.

This plot shows the electronic of iron-decorated graphene (Fe-C). The TDOS (black line), with the Fermi level near -5 eV is mainly shaped by the graphene PDOS (red line), featuring peaks around -10 eV, -2 eV and 6 eV. The highlights graphene's dominant role in determining the overall electronic properties of the Fe-C system.

The PDOS of iron (blue line) shows small, localized peaks around 3 eV, indicating limited hybridization with graphene and a weaker overall contribution to the total density of states. The OPDOS (green line) reveals minor contributions in the deep valance band (-22 to 10 eV) likely from specific iron or carbon orbitals involved in their interaction. Overall, the plot highlights graphene's dominant electronic character in the Fe-C system, with iron adding localized states that may affect its magnetic or catalytic properties.

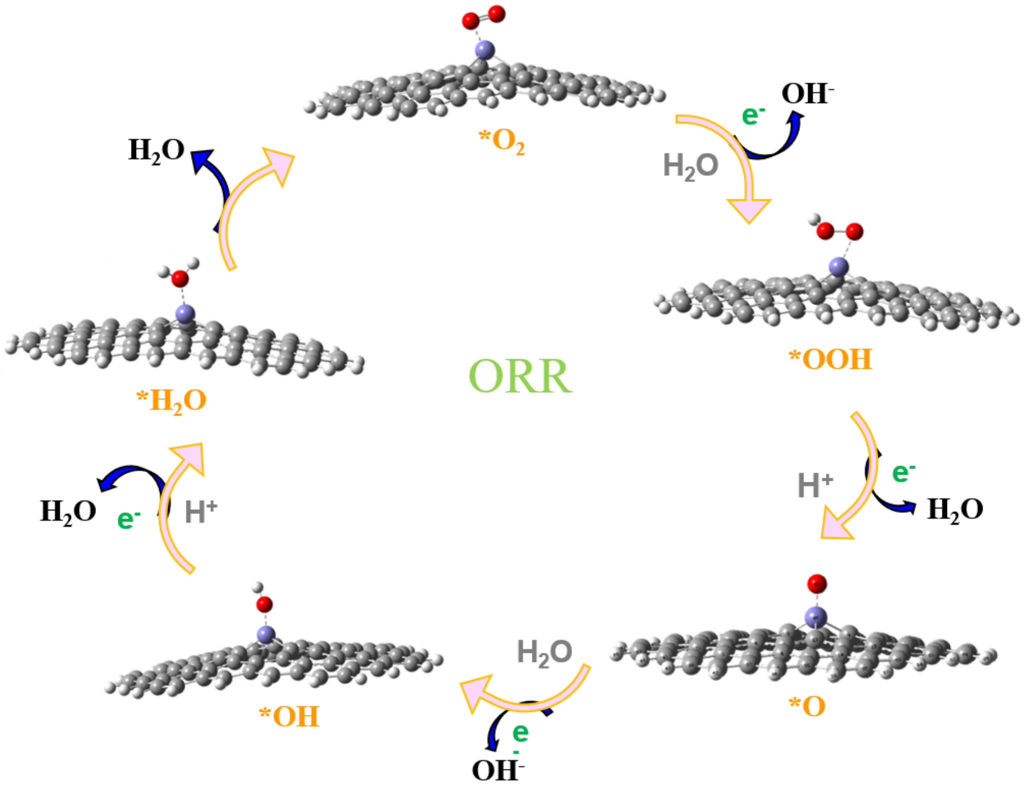


**Figure S24.** Schematic illustration of ORR mechanism at the Fe active site of Fe NPs@PC compound and their adsorption intermediates (*$O_2$, *OOH, *O *OH, and*$H_2O$).

**Model Fe clusters of different sizes (1-3 nm vs 5-7 nm)**

We have modeled with 3Fe and 5Fe on graphitic carbon framework to mimic the 3 and 5 nm Fe clusters observed in TEM. Various oxygen-containing species such as O, $O_2$, OH, OOH, and $H_2O$ were allowed to interact with the 3Fe and 5Fe active sites of the graphitic carbon framework, and density functional theory (DFT) calculations were performed at HF level of theory in the gas phase using the Gaussian 16[3] software package. The HF method is an ab initio approach that solves the Schrödinger equation using a mean-field approximation, each electron feels the average effect of all other electrons. HF neglects electron correlation beyond exchange interactions, which leads to more approximate total energies and molecular properties especially for systems with significant electron correlation such as hydrogen bonding. Due to the time limitation and computational cost. the HF method has been employed. The B3LYP (Becke, three-parameter, Lee-yang-Parr)[4] method were utilized for single point energy calculation to run the frontier molecular orbital calculations.[5] This method allows us to capture both exchange and dynamic correlation effects, offering improved accuracy for electronic properties. The bond length of Fe…C and Fe….O, $O_2$, OH, $H_2O$, OOH, (for all reaction coordinates) for all the optimized structures were depicted in **Figure S27**. It can be observed that the Fe…C bond length has been highly influenced by the electronegativity of the oxygen atom from the reaction coordinates in case of both 3 and 5 nm Fe clusters.

To understand the interactions between 3Fe and 5Fe on graphitic carbon framework and adsorbed species, the binding energies were calculated using density functional theory (DFT) see in **Figure 28**. The considered adsorbates include $H_2O$, O, $O_2$, OH, and OOH, the corresponding

binding energies are expressed in electron volts (eV). For 3Fe cluster, the calculated binding energies are -2.78 eV ($H_2O$), -7.833 eV (O), -5.347 eV ($O_2$), -35.745 eV (OH), and -28.864 eV (OOH). Among these, the OH species observed as the strongest adsorption and the high stability. Similarly, in 5Fe cluster, the binding energies are -2.345 eV ($H_2O$), -9.066 eV (O), -4.672 eV ($O_2$), -42.253 eV (OH), and -14.214 eV (OOH). The result clearly shows that in both clusters the hydroxyl group adsorb strongly. This interaction with OH species highlights their potential as promising catalyst.

**Density of states (DOS) analysis:**

To gain insight into the influence of surface functionalization and oxygen species on the electronic properties of 3Fe, 5Fe clusters TDOS and PDOS were employed at B3LYP/6-31g(d)//HF level in the gas phase for pristine and oxygenated systems using Multiwfn.[6] The Fermi level ($E_n$ = 0 a.u.) serves as the reference. In all cases, graphene (red line) and Fe (blue line) contribute dominantly to the total electronic structure, while oxygen-derived species (pink line) introduce distinct modifications depending on their chemical state.

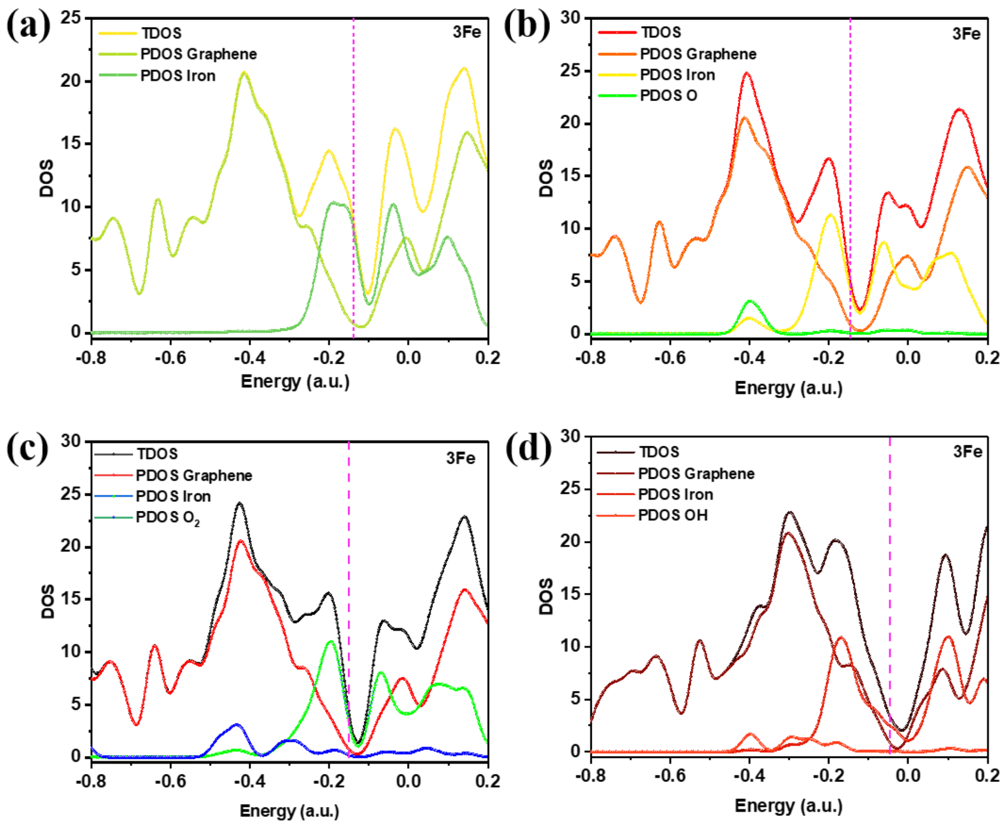


**Figure S25.** (a) Total Density of states (TDOS) and Partial Density of States of 3Fe cluster employed at B3LYP/6-31g(d)//HF level in the gas phase. (b) Total Density of states (TDOS) and Partial Density of States of Oxygen adsorbed on 3Fe cluster-Graphene system. (c) Total Density of states (TDOS) and Partial Density of States of $O_2$ adsorbed 3Fe-graphene cluster. (d) Total Density of states (TDOS) and Partial Density of States of OH adsorbed 3Fe-graphene cluster.

**3Fe System:**

The **Figure 25a** presents the calculated Density of States for a system comprising graphene, and iron of 3Fe-Graphene system. The plot displays the total density of states (TDOS) in black, the partial density of states (PDOS) projected onto graphene (red), and iron(blue). The energy is plotted in atomic units (a.u) relative to the Fermi level, which is indicated by the vertical dashed line at approximately -0.10 eV and y-axis represents the density of states.

The TDOS (black line) displays significant intensity in both the valence and conduction regions,

confirming the metallic nature of the system. The graphene PDOS closely follows the TDOS profile, particularly in the regions around –0.45 a.u and 0.10 a.u indicating the carbon $\pi$ and $\pi^*$ orbitals dominate the overall electronic contribution. The Fe PDOS exhibits smaller, localized peaks centered near –0.30 a.u and slightly below the Fermi level, suggesting partial hybridization. The finite TDOS at the Fermi level implies that charge transfer between Fe and graphene occurs, maintaining electronic conductivity and confirming Fe as an active site for adsorption or redox reactions.

This **Figure 25b** presents the calculated Density of States for a system comprising graphene, and iron. The plot displays the total density of states (TDOS) in black, the partial density of states (PDOS) projected onto graphene (red), iron(blue) and oxygen (Pink). The energy is plotted in atomic units (a.u) relative to the Fermi level, which is indicated by the vertical dashed line at approximately-0.10eV and y-axis represents the density of states.

The interaction between atomic oxygen and 3Fe produces marked modifications in the DOS pattern. The TDOS shows a pronounced valley just below the Fermi level, indicating partial localization of electronic states due to strong Fe-O bonding. The oxygen PDOS (pink line) exhibits sharp peaks centered around -0.40 a.u due to overlapping, signifying strong hybridization and the formation of Fe-O bonds. The graphene PDOS intensity near the Fermi level decreases further, indicating charge transfer from graphene to the Fe-O complex. Such strong hybridization is typically associated with high catalytic activity for oxidation reactions, as it facilitates O-Fe charge exchange during reaction cycles.

The **Figure 25c** presents the calculated Density of States for a system comprising $O_2$ on 3Fe-graphene system. The plot displays the total density of states (TDOS) in black, the partial density of states (PDOS) projected onto graphene (red), iron(blue) and $O_2$ (Pink). The energy is plotted

in atomic units (a.u) relative to the Fermi level, which is indicated by the vertical dashed line at approximately-0.10eV and y-axis represents the density of states.

The $O_2$ PDOS (pink line) shows low-intensity, broad features between -0.45 a.u and -0.30 a.u corresponding to partially filled $\pi^*$ orbitals of $O_2$ that weakly hybridize with Fe 3d states. The partial overlap with Fe PDOS indicates slight charge transfer. The TDOS at the Fermi level indicates that the metallic nature of the $O_2$ adsorbed 3Fe substrate remains intact. These observations suggest that Fe centers on graphene can activate $O_2$ molecules through electron donation into their antibonding orbitals, an essential step in oxygen reduction and catalytic oxidation reactions.

The **Figure 25d** presents the calculated Density of States for a system comprising O, graphene, and iron. The plot displays the total density of states (TDOS) in black, the partial density of states (PDOS) projected onto graphene (red), iron(blue) and OH (Pink). The energy is plotted in atomic units (a.u) relative to the Fermi level, which is indicated by the vertical dashed line at approximately -0.10 eV and y-axis represents the density of states

Upon OH adsorption, the TDOS maintains its general shape but exhibits a modest redistribution of states near the Fermi level. A distinct low-energy feature emerges in the PDOS of the hydroxyl group (pink line) between -0.40 a.u and -0.30 a.u indicating the presence of localized O-H bonding states. The Fe PDOS shows increased intensity overlapping this region, confirming orbital hybridization. The graphene PDOS still dominates the total DOS but shows a slight reduction near the Fermi level, suggesting that electron density is partially drawn toward the adsorbed OH group. These changes reveal that OH adsorption leads to charge polarization at the Fe site, slightly lowering the metallic character while introducing reactive surface states relevant for catalytic reactions.

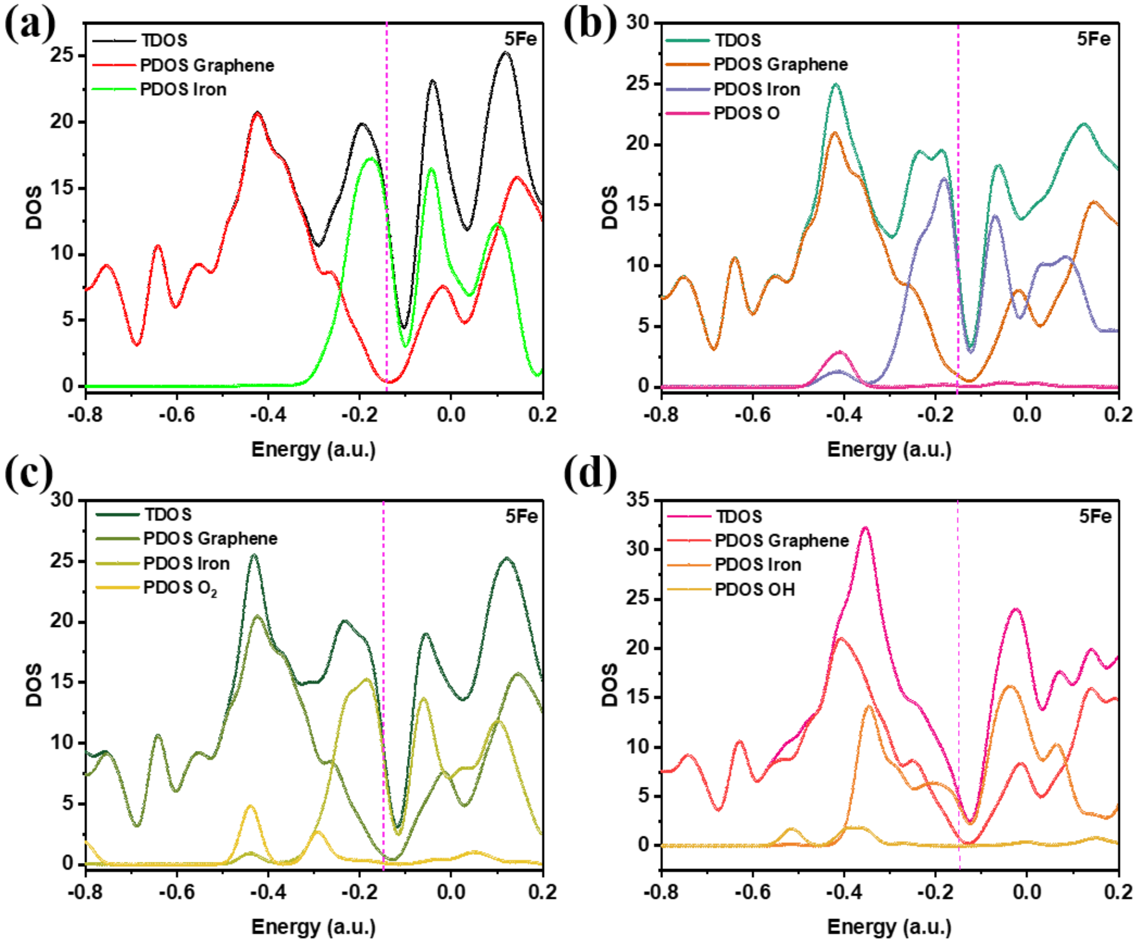


**Figure S26.** (a) Total Density of states (TDOS) and Partial Density of States of 5Fe-graphene cluster. (b) Total Density of states (TDOS) and Partial Density of States of oxygen adsorbed 5Fe-graphene cluster. (c) Total Density of states (TDOS) and Partial Density of States of $O_2$ adsorbed 5Fe-graphene cluster. (d) Total Density of states (TDOS) and Partial Density of States of OH adsorbed 5Fe-graphene cluster.

**5Fe System:**

The **Figure 26a** presents the calculated Density of States for a system comprising graphene, and iron. The plot displays the total density of states (TDOS) in black, the partial density of states (PDOS) projected onto graphene (red), and iron(blue). The energy is plotted in atomic units (a.u)

relative to the Fermi level, which is indicated by the vertical dashed line at approximately-0.10eV and y-axis represents the density of states.

The TDOS of the 5Fe (black line) exhibits significant electronic states both below and above the Fermi level, confirming its metallic nature. The Fermi level is located near -0.10 a.u. The PDOS of graphene (red line) dominates throughout the valence region, particularly between -0.60 a.u and -0.30 a.u indicating that the carbon is the primary contributor to the total states. Iron (blue line) shows notable peaks overlapping with those of graphene, especially around -0.40 a.u suggesting hybridization. This hybridization implies moderate Fe-C bonding interactions, stabilizing the Fe anchoring site on graphene and promoting electronic communication between Fe centers and the support.

The **Figure 26b** presents the calculated Density of States for a system comprising O, graphene, and iron. The plot displays the total density of states (TDOS) in black, the partial density of states (PDOS) projected onto graphene (red), iron(blue) and Oxygen (Pink). The energy is plotted in atomic units (a.u) relative to the Fermi level, which is indicated by the vertical dashed line at approximately-0.10eV and y-axis represents the density of states.

In the case of oxygen adsorption, a distinct PDOS contribution from oxygen (pink) emerges near -0.45 a.u overlapping with Fe 3d states. The strong alignment of Fe and O peaks in this region confirms significant Fe-O orbital mixing. Compared to other adsorbates, the hybridization here is more pronounced, and a noticeable shift of TDOS intensity toward lower energies. These spectral changes indicate stronger Fe-O bond formation and a redistribution of charge density. The system retains metallic features, but the Fe-O interaction leads to partial electronic localization that could influence the catalytic activity at the Fe site.

The **Figure 26c** presents the calculated Density of States for a system comprising $O_2$, graphene,

and iron. The plot displays the total density of states (TDOS) in black, the partial density of states (PDOS) projected onto graphene (red), iron(blue) and $O_2$ (Pink). The energy is plotted in atomic units (a.u) relative to the Fermi level, which is indicated by the vertical dashed line at approximately -0.10eV and y-axis represents the density of states.

For the $O_2$ adsorbed configuration, the PDOS of oxygen (pink) reveals two distinct features between -0.45 a.u and -0.25 a.u corresponding to π* orbitals of the adsorbed $O_2$ molecule. These peaks overlap with Fe, confirming orbital hybridization. The overlap intensity and broadening of Fe peaks indicate the formation of Fe-$O_2$ co-ordination bonds and possible charge transfer from Fe to the antibonding orbitals of $O_2$. The TDOS near the Fermi level remains non-zero, confirming metallic behavior.

The **Figure 26d** presents the calculated Density of States for a system comprising OH, graphene, and iron. The plot displays the total density of states (TDOS) in black, the partial density of states (PDOS) projected onto graphene (red), iron(blue) and OH(Pink). The energy is plotted in atomic units (a.u) relative to the Fermi level, which is indicated by the vertical dashed line at approximately-0.10eV and y-axis represents the density of states.

Upon OH adsorption, new localized states appear in the valence region around -0.50 a.u (pink line). This peak partially overlaps with Fe 3d states, signifying Fe-O hybridization and the formation of Fe-OH bonds. The intensity of the Fe PDOS near the Fermi level decreases slightly compared with 5Fe, indicating partial charge redistribution from Fe to O. This electronic rearrangement suggests a stronger Fe-O bond and a slightly reduced metallic character upon OH attachment.

| Catalyst + Reaction coordinates (RC) | 1-3 nm Sized Fe Cluster | 5-7 nm Sized Fe Cluster |
|---|---|---|
| **Pristine Catalyst** | 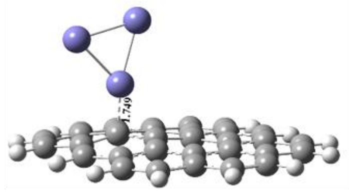 | 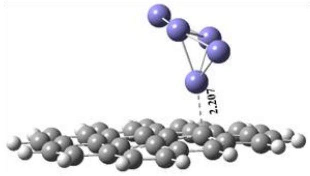 |
| **Catalyst + $O_2$** | 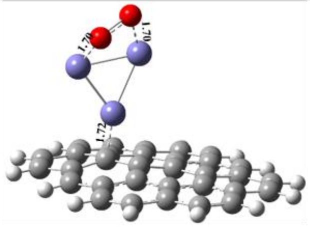 | 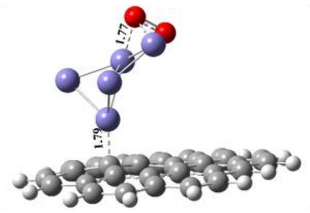 |
| **Catalyst + OOH** | 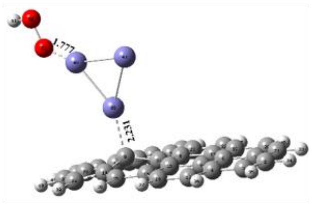 | 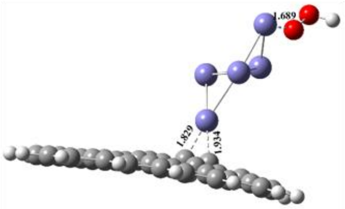 |
| **Catalyst + O** | 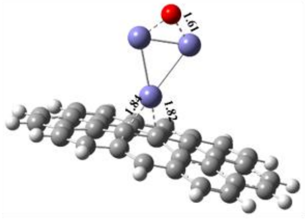 | 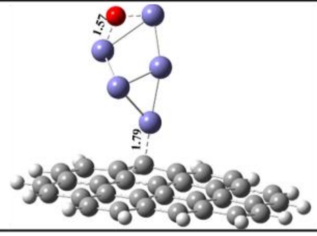 |
| **Catalyst + OH** | 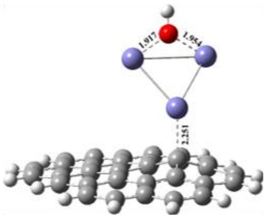 | 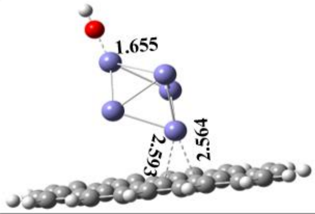 |
| **Catalyst + $H_2O$** | 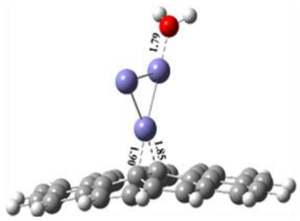 | 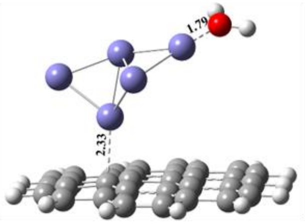 |

**Figure S27.** Optimized structure of 1-3 nm and 5-7 nm sized Fe clusters.

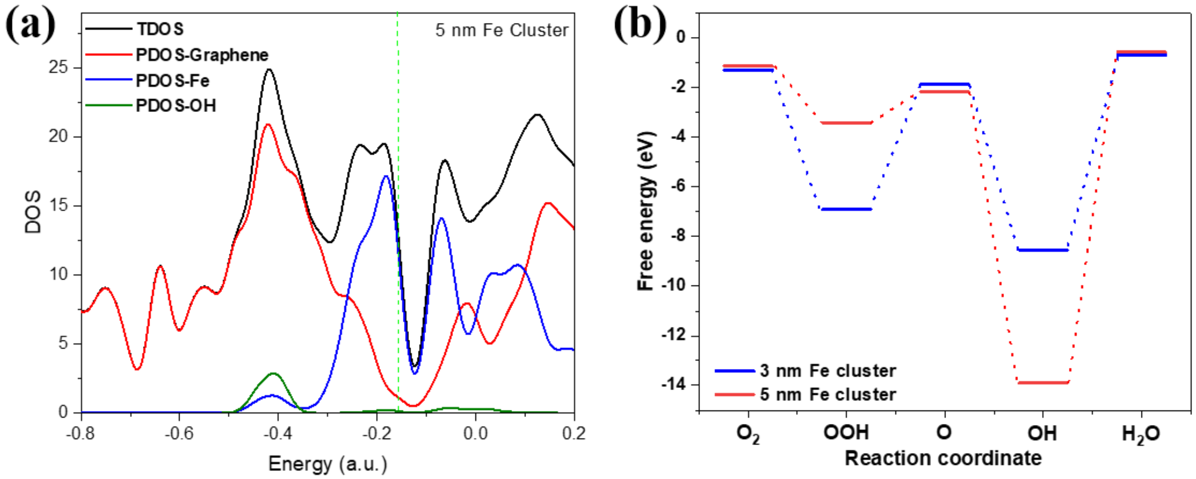


**Figure S28.** (a) DOS and TDOS, (b) free energy diagram of 3 nm and 5 nm Fe clusters.

**Table S1.** Comparison table of MOF-derived iron-carbon based electrocatalysts for OER/ORR (overpotential/half wave potential) and Zn-air battery (power density) performances.

| S. No | Catalysts | Half-wave potential ($E_{1/2}$, V) | Overpotential (OER $\eta_{10}$, mV) | Power density (mW $cm^{-2}$)/OCV | Year | Ref. |
|---|---|---|---|---|---|---|
| 1 | Fe NPs@PC-700 | 0.786 | 320 | 219 | 2025 | **This work** |
| 2 | $Ni_{0.6}Fe_{0.4}$ | 0.75 | 280 | 59.83 | 2022 | [7] |
| 3 | FeS/$Fe_3C$@NSC | 0.78 | 270 | 90.9/1.455 | 2020 | [8] |
| 4 | Fe-HCNS | 0.76 | 450 | - | 2024 | [9] |
| 5 | POP-Fe/Ni-900 | 0.82 | 390 | 256/1.40 | 2023 | [10] |
| 6 | NiFe -NC | 0.81 | 430 | 78.8/1.38 | 2024 | [11] |
| 7 | Fe-NPC-1000 | 0.79 | 420 | 55.1/1.3 | 2021 | [12] |
| 8 | $Ni_3FeN$/NRGO | 0.72 | 400 | 1.3 | 2017 | [13] |
| 9 | Ni-Fe-MoN NTs | 0.72 | 300 | 118/1.4 | 2018 | [14] |
| 10 | $Ni_3Fe$/N-C sheet | 0.76 | 370 | 1.3 | 2016 | [15] |
| 11 | CoFe@CNT | 0.85 | 370 | 115/1.5 | 2025 | [16] |
| 12 | Fe–Zn@NCF | 0.88 | 352 | 184.6/1.5 | 2022 | [17] |
| 13 | FeNi/NC | 0.83 | 410 | 80.8 | 2018 | [18] |
| 14 | Fe/Co-NGr | 0.78 | 400 | 149 | 2024 | [19] |
| 15 | FeCo/$Co_2P$@NPCF | 0.79 | 330 | 154 | 2020 | [20] |
| 16 | $Co_2FeO_4$/NCNTs | 0.89 | 370 | 150 | 2020 | [21] |

**Table S2.** Comparison table of metal-carbon based electrocatalysts for Zn-air battery (power density) performances.

| S. No | Catalysts | Open circuit potential (OCV) | Power density (mW $cm^{-2}$) | Specific capacity (mA h $g^{-1}$) | Year | Ref. |
|---|---|---|---|---|---|---|
| 1 | Fe NPs@PC-700 | 1.49 | 219 | 754 | 2025 | **This work** |
| 2 | FeNi/N-GPCM | 1.473 | 321 | 761.2 | 2023 | [22] |
| 3 | CoO/N-CNT | 1.40 | 265 | 570 | 2013 | [23] |
| 4 | FeNi@NC | 1.45 | 116 | - | 2023 | [24] |
| 5 | FeOCo-SAD | 1.52 | 241.24 | 820 | 2024 | [25] |
| 6 | FeMn─N─C | 1.494 | 151 | 795 | 2024 | [26] |
| 7 | Fe,Co,N–C | 1.40 | 198.4 | 726 | 2022 | [27] |
| 8 | FeCo-NSC | 1.49 | 152.8 | 782.1 | 2022 | [28] |
| 9 | (Fe,Co)-SA/CS | 1.43 | 86.65 | 819.6 | 2020 | [29] |
| 10 | FeCo-NCNTs | 1.55 | 148 | - | 2021 | [30] |
| 11 | Fe–Ni ANC@NSC | 1.42 | 140.3 | 750.7 | 2021 | [31] |
| 12 | FeNi-NPC | 1.48 | 226.5 | 661 | 2023 | [32] |
| 13 | FC-C@NC | 1.466 | 118.2 | 659.5 | 2020 | [33] |
| 14 | Fe/Cu-N-C | 1.48 | 183 | 805 | 2023 | [34] |
| 15 | Fe/Cu-N-C | 1.50 | 116.6 | 809.2 | 2024 | [35] |
| 16 | $Fe_3O_4$/CuNCs/ZnNx | 1.45 | 162 | 760 | 2024 | [36] |

**References**

[1] R.S. Kumar, S. Prabhakaran, S. Ramakrishnan, S.C. Karthikeyan, A.R. Kim, D.H. Kim, D.J. Yoo, Developing Outstanding Bifunctional Electrocatalysts for Rechargeable Zn-Air Batteries Using High-Purity Spinel-Type ZnCo2Se4 Nanoparticles, Small, 19 (2023) 2207096.

[2] R.S. Kumar, S. Tamilarasi, A.M. Stephan, A.R. Kim, D.J. Yoo, CrS Doped MOF-Derived Carbon Implanted CoNi Particles as Exceedingly Effectual Oxygen Electrocatalysts in Sustainable Zinc-Air Batteries, Small Methods, n/a (2025) 2401515.

[3] M.J. Frisch, G.W. Trucks, H.B. Schlegel, G.E. Scuseria, M.A. Robb, J.R. Cheeseman, G. Scalmani, V. Barone, G.A. Petersson, H. Nakatsuji, X. Li, M. Caricato, A.V. Marenich, J. Bloino, B.G. Janesko, R. Gomperts, B. Mennucci, H.P. Hratchian, J.V. Ortiz, A.F. Izmaylov, J.L. Sonnenberg, Williams, F. Ding, F. Lipparini, F. Egidi, J. Goings, B. Peng, A. Petrone, T. Henderson, D. Ranasinghe, V.G. Zakrzewski, J. Gao, N. Rega, G. Zheng, W. Liang, M. Hada, M. Ehara, K. Toyota, R. Fukuda, J. Hasegawa, M. Ishida, T. Nakajima, Y. Honda, O. Kitao, H. Nakai, T. Vreven, K. Throssell, J.A. Montgomery Jr., J.E. Peralta, F. Ogliaro, M.J. Bearpark, J.J. Heyd, E.N. Brothers, K.N. Kudin, V.N. Staroverov, T.A. Keith, R. Kobayashi, J. Normand, K. Raghavachari, A.P. Rendell, J.C. Burant, S.S. Iyengar, J. Tomasi, M. Cossi, J.M. Millam, M. Klene, C. Adamo, R. Cammi, J.W. Ochterski, R.L. Martin, K. Morokuma, O. Farkas, J.B. Foresman, D.J. Fox, Gaussian 16 Rev. C.01, Wallingford, CT, 2016.

[4] A.D. Becke, Density-functional thermochemistry. III. The role of exact exchange, The Journal of Chemical Physics, 98 (1993) 5648-5652.

[5] A. Szabo, N.S. Ostlund, Modern Quantum Chemistry: Introduction to Advanced Electronic Structure Theory, Dover Publications1996.

[6] T. Lu, F. Chen, Multiwfn: A multifunctional wavefunction analyzer, Journal of Computational

Chemistry, 33 (2012) 580-592.

[7] Y.-y. Li, Q. Zou, Z. Li, D. Xie, Y. Niu, J. Zou, X. Zeng, J. Huang, MOF derived Ni-Fe based alloy carbon materials for efficient bifunctional electrocatalysts applied in Zn-air battery, Applied Surface Science, 572 (2022) 151286.

[8] Y.-W. Li, W.-J. Zhang, J. Li, H.-Y. Ma, H.-M. Du, D.-C. Li, S.-N. Wang, J.-S. Zhao, J.-M. Dou, L. Xu, Fe-MOF-Derived Efficient ORR/OER Bifunctional Electrocatalyst for Rechargeable Zinc–Air Batteries, ACS Applied Materials & Interfaces, 12 (2020) 44710-44719.

[9] Z. Ji, C. Qiu, P. Li, Y. Li, J. Shi, J. Wu, Y. Zhu, W. Hu, Z. Li, H. Wang, Atomic modulation of FeN4 sites on hollow carbon nanospheres by neighboring Mn atoms for ultra-stable Zn-air batteries, Chemical Engineering Journal, 500 (2024) 157505.

[10] P. Weng, Y. Guo, K. Wu, X. Wang, G.-Q. Huang, H. Lei, Y. Yuan, W. Lu, D. Li, Design of Fe/Ni-doped N/S-rich carbon with advanced bifunctional electrocatalysis for Zn–air batteries, Journal of Materials Chemistry A, 11 (2023) 12194-12201.

[11] B. Ricciardi, W. da Silva Freitas, B. Mecheri, K.U. Nisa, J. Montero, V.C.A. Ficca, E. Placidi, C. Alegre, A. D'Epifanio, Hierarchical porous Fe/Ni-based bifunctional oxygen electrocatalysts for rechargeable zinc-air batteries, Carbon, 219 (2024) 118781.

[12] S. Han, Y. Ding, Q. Qian, L. Ruan, X. Wang, Y. Zhu, M. Zhu, Highly active electrocatalyst for rechargeable Zn-air battery: 3D Fe/N-based honeycomb-like carbon, Vacuum, 188 (2021) 110201.

[13] Y. Fan, S. Ida, A. Staykov, T. Akbay, H. Hagiwara, J. Matsuda, K. Kaneko, T. Ishihara, Ni-Fe Nitride Nanoplates on Nitrogen-Doped Graphene as a Synergistic Catalyst for Reversible Oxygen Evolution Reaction and Rechargeable Zn-Air Battery, Small, 13 (2017) 1700099.

[14] C. Zhu, Z. Yin, W. Lai, Y. Sun, L. Liu, X. Zhang, Y. Chen, S.-L. Chou, Fe-Ni-Mo Nitride

Porous Nanotubes for Full Water Splitting and Zn-Air Batteries, Advanced Energy Materials, 8 (2018) 1802327.

[15] G. Fu, Z. Cui, Y. Chen, Y. Li, Y. Tang, J.B. Goodenough, Ni3Fe-N Doped Carbon Sheets as a Bifunctional Electrocatalyst for Air Cathodes, Advanced Energy Materials, 7 (2017) 1601172.

[16] Z. Fu, H. Zhuo, X. Liu, W. Li, H. Song, Z. Shi, L. Feng, T. Jin, W. Chen, Y. Chen, Fe/Co Bimetal-Containing Carbon Prepared from a 2D Metalloporphyrin-Based MOF for the Optimal ORR/OER Bifunction and Its Application in Zn–Air Batteries, ACS Applied Energy Materials, 8 (2025) 1051-1059.

[17] Z. Zhu, P. Liu, P. Du, B. Yu, X. Li, Y. Wang, L.-P. Lv, Fe, Zn Co-Doped Porous Carbon Nanofiber-Based Rechargeable Zinc Air Batteries with Stable Operation over 1600 h, Industrial & Engineering Chemistry Research, 62 (2023) 169-179.

[18] L. Yang, X. Zeng, D. Wang, D. Cao, Biomass-derived FeNi alloy and nitrogen-codoped porous carbons as highly efficient oxygen reduction and evolution bifunctional electrocatalysts for rechargeable Zn-air battery, Energy Storage Materials, 12 (2018) 277-283.

[19] M. Mooste, Z. Ahmed, P. Kapitulskis, R. Ivanov, A. Treshchalov, H.-M. Piirsoo, A. Kikas, V. Kisand, K. Kukli, I. Hussainova, K. Tammeveski, Bifunctional oxygen electrocatalyst based on Fe, Co, and nitrogen co-doped graphene-coated alumina nanofibers for Zn-air battery air electrode, Applied Surface Science, 660 (2024) 160024.

[20] Q. Shi, Q. Liu, Y. Ma, Z. Fang, Z. Liang, G. Shao, B. Tang, W. Yang, L. Qin, X. Fang, High-Performance Trifunctional Electrocatalysts Based on FeCo/Co2P Hybrid Nanoparticles for Zinc–Air Battery and Self-Powered Overall Water Splitting, Advanced Energy Materials, 10 (2020) 1903854.

[21] X. Duan, S. Ren, N. Pan, M. Zhang, H. Zheng, MOF-derived Fe,Co@N–C bifunctional

oxygen electrocatalysts for Zn–air batteries, Journal of Materials Chemistry A, 8 (2020) 9355-9363.

[22] M. Zhang, X. Hu, Y. Xin, L. Wang, Z. Zhou, L. Yang, J. Jiang, D. Zhang, FeNi coordination polymer based highly efficient and durable bifunction oxygen electrocatalyst for rechargeable zinc-air battery, Separation and Purification Technology, 308 (2023) 122974.

[23] Y. Li, M. Gong, Y. Liang, J. Feng, J.-E. Kim, H. Wang, G. Hong, B. Zhang, H. Dai, Advanced zinc-air batteries based on high-performance hybrid electrocatalysts, Nature Communications, 4 (2013) 1805.

[24] L. Duan, Z. Ren, X. Chen, D. Zhang, S. Xu, FeNi Confined in N-Doped Carbon as a Highly Efficient Bi-Functional Catalyst for Rechargeable Zn–Air Batteries, Inorganics, 2023.

[25] Q. Zhou, W. Xue, X. Cui, P. Wang, S. Zuo, F. Mo, C. Li, G. Liu, S. Ouyang, S. Zhan, J. Chen, C. Wang, Oxygen-bridging Fe, Co dual-metal dimers boost reversible oxygen electrocatalysis for rechargeable Zn–air batteries, Proceedings of the National Academy of Sciences, 121 (2024) e2404013121.

[26] C. Hu, G. Xing, W. Han, Y. Hao, C. Zhang, Y. Zhang, C.-H. Kuo, H.-Y. Chen, F. Hu, L. Li, S. Peng, Inhibiting Demetalation of Fe─N─C via Mn Sites for Efficient Oxygen Reduction Reaction in Zinc-Air Batteries, Advanced Materials, 36 (2024) 2405763.

[27] S. Sarkar, A. Biswas, E.E. Siddharthan, R. Thapa, R.S. Dey, Strategic Modulation of Target-Specific Isolated Fe,Co Single-Atom Active Sites for Oxygen Electrocatalysis Impacting High Power Zn–Air Battery, ACS Nano, 16 (2022) 7890-7903.

[28] Y. Wu, C. Ye, L. Yu, Y. Liu, J. Huang, J. Bi, L. Xue, J. Sun, J. Yang, W. Zhang, X. Wang, P. Xiong, J. Zhu, Soft template-directed interlayer confinement synthesis of a Fe-Co dual single-atom catalyst for Zn-air batteries, Energy Storage Materials, 45 (2022) 805-813.

[29] V. Jose, H. Hu, E. Edison, W. Manalastas Jr, H. Ren, P. Kidkhunthod, S. Sreejith, A. Jayakumar, J.M.V. Nsanzimana, M. Srinivasan, J. Choi, J.-M. Lee, Modulation of Single Atomic Co and Fe Sites on Hollow Carbon Nanospheres as Oxygen Electrodes for Rechargeable Zn–Air Batteries, Small Methods, 5 (2021) 2000751.

[30] S.-Y. Lin, L.-X. Xia, L. Zhang, J.-J. Feng, Y. Zhao, A.-J. Wang, Highly active Fe centered FeM-N-doped carbon (M = Co/Ni/Mn): A general strategy for efficient oxygen conversion in Zn–air battery, Chemical Engineering Journal, 424 (2021) 130559.

[31] H. Li, X. Shu, P. Tong, J. Zhang, P. An, Z. Lv, H. Tian, J. Zhang, H. Xia, Fe–Ni Alloy Nanoclusters Anchored on Carbon Aerogels as High-Efficiency Oxygen Electrocatalysts in Rechargeable Zn–Air Batteries, Small, 17 (2021) 2102002.

[32] Z. Wang, C. Li, Y. Liu, Y. Wu, S. Zhang, C. Deng, Atomically dispersed Fe-Ni dual sites in heteroatom doped carbon tyres for efficient oxygen electrocatalysis in rechargeable Zn-Air battery, Journal of Energy Chemistry, 83 (2023) 264-274.

[33] K. Zhang, Y. Zhang, Q. Zhang, Z. Liang, L. Gu, W. Guo, B. Zhu, S. Guo, R. Zou, Metal-organic framework-derived Fe/Cu-substituted Co nanoparticles embedded in CNTs-grafted carbon polyhedron for Zn-air batteries, Carbon Energy, 2 (2020) 283-293.

[34] M. Bu, Y. Liu, S. Liao, W. Liu, Z. Yang, J. Jiang, X. Gao, Y. Yang, H. Liu, In-site grown carbon nanotubes connecting Fe/Cu-N-C polyhedrons as robust electrocatalysts for Zn-air batteries, Carbon, 214 (2023) 118365.

[35] W. Zhang, B. Feng, L. Huang, Y. Liang, J. Chen, X. Li, Z. Shi, N. Wang, Fe/Cu diatomic sites dispersed on nitrogen-doped mesoporous carbon for the boosted oxygen reduction reaction in Mg-air and Zn-air batteries, Applied Catalysis B: Environment and Energy, 358 (2024) 124450.

[36] Y. Pan, Q. Yang, X. Liu, F. Qiu, J. Chen, M. Yang, Y. Fan, H. Song, S. Zhang, Multi-metal (Fe, Cu, and Zn) coordinated hollow porous dodecahedron nanocage catalyst for oxygen reduction in Zn–air batteries, Energy Advances, 3 (2024) 2648-2657.

S
